# OPG-Producing B Cells and RANKL-Expressing T Cells Define Immune Signatures Predictive of Bone Metastases in Breast Cancer

**DOI:** 10.1158/2767-9764.CRC-25-0696

**Published:** 2026-01-13

**Authors:** Ana Carolina Monteiro, Diego D. Garcia, Ana Paula A. Fontão, Bárbara Du Rocher, Maria E. Globa Masset, Isabella N. Alves, Lucas Gonçalves Carvalho, Cecília Vianna de Andrade, Gabriel Fidalgo, Marcos Vinicius Colaço, Liebert Parreiras Nogueira, Maria de Fatima Dias Gaui, Adriana Bonomo

**Affiliations:** 1 Laboratory of Osteo and Tumor Immunology, Department of Immunobiology, Fluminense Federal University, Niterói, Brazil.; 2 Laboratory on Thymus Research, Rio de Janeiro, Brazil.; 3 Department of Pathology, Instituto Fernandes Figueira, Oswaldo Cruz Foundation, Rio de Janeiro, Brazil.; 4 Laboratory of Applied Physics to Biomedical and Environmental Sciences, Physics Institute, State University of Rio de Janeiro, Rio de Janeiro, Brazil.; 5 Oral Research Laboratory, Institute of Clinical Dentistry, University of Oslo, Oslo, Norway.; 6 Internal Medicine, Federal University of Rio de Janeiro, Rio de Janeiro, Brazil.; 7 National Institute of Science and Technology on Neuroimmunomodulation (INCT-NIM), Rio de Janeiro, Brazil.; 8 Research Network on Neuroinflammation (RENEURIN), Oswaldo Cruz Institute, Rio de Janeiro, Brazil.

## Abstract

Bone metastases represent a common and devastating complication of breast cancer; however, the immune determinants of skeletal colonization, particularly the role of adaptive lymphocyte subsets, have only recently been elucidated. In this study, we identified a nonclassical regulatory role of CD19^+^ B cells in the modulation of bone remodeling and breast cancer metastatic progression. Using murine models of metastatic 4T1 and nonmetastatic 67NR mammary tumors, we show that 67NR-bearing mice exhibit increased trabecular bone mass, associated with osteoprotegerin (OPG) secretion by CD19^+^IgD^+^IgM^+^CD138^−^ B cells. Functional assays demonstrated that 67NR-primed B cells suppressed bone resorption *in vitro* and counteracted 4T1-specific T cell–mediated osteolytic activity *in vivo*. Adoptive transfer of 67NR-primed CD19^+^ B cells into 4T1-bearing immunocompetent or immunodeficient hosts preserved bone architecture, reduced RANKL production, inhibited metastases, and limited tumor growth. These effects are T cell–independent at the effector phase but require T cell-licensing for the acquisition of the OPG-producing phenotype. Silencing OPG abrogated this protective function. Mechanistically, B cells must be transferred during the early stages of tumor progression to retain their therapeutic potential. Molecular analyses supported the enrichment of RANKL^+^CD4^+^ T cells in tumors and lymph nodes, whereas OPG^+^ B cells were restricted to tumors. Transcriptomic data from The Cancer Genome Atlas further support the prognostic relevance of these immune phenotypes. In a retrospective human cohort, high RANKL^+^ lymphocyte infiltration in primary tumors correlated with bone metastases, whereas OPG^+^ infiltration predominated in bone metastasis–free cases. These findings uncover a B cell–driven axis that restrains osteolysis and tumor progression.

**Significance:**

This study identifies opposing RANKL^+^ T cell and OPG-producing B cell immune phenotypes that shape bone metastasis risk in breast cancer. By revealing how these adaptive lymphocyte subsets influence osteolysis and skeletal colonization, our findings define prognostic immune signatures with potential utility for early risk stratification and clinical decision-making.

## Introduction

The bone marrow (BM) is a frequent site of breast cancer metastasis and contributes substantially to patient morbidity and mortality worldwide (1). Despite therapeutic advances, current treatments remain largely palliative, failing to disrupt the vicious osteolytic cycle or to prevent metastatic seeding and outgrowth (1). This therapeutic gap underscores the need to elucidate the cellular and molecular mechanisms governing tumor-bone interactions, with the aim of developing new targeted strategies.

Skeletal homeostasis relies on tightly regulated bone remodeling orchestrated by the RANK–RANKL–osteoprotegerin (OPG) axis (2). This molecular signaling system maintains a balance between bone formation by osteoblasts (OB) and bone resorption by osteoclasts (OC; refs. 2–4). Under physiologic conditions, receptor activator of NF-κB ligand (RANKL, encoded by TNFSF11) binds to its receptor, RANK (TNFRSF11A), on OC precursors, promoting their differentiation into mature functional OCs (3). Following bone resorption, OB-lineage cells secrete OPG (TNFRSF11B), a decoy receptor that binds to RANKL, thereby inhibiting its interaction with RANK and halting OC activity to preserve skeletal integrity (4). *In situ* hybridization studies have shown that the spatial distribution of RANKL and OPG within bone tissue is highly organized, with osteoprogenitor cells along bone surfaces as the primary source of RANKL, whereas osteocytes and OBs serve as the main producers of OPG (5). Recent findings emphasize that the protective effect of OPG depends not on its systemic concentration but rather on its local production in response to cytokines, such as TGFβ-1, released during bone resorption (6). These results strongly suggest that circulating OPG alone is insufficient to compensate for local deficiencies, underscoring the critical role of tissue-resident or migrating cells within the BM in preserving skeletal integrity (6).

In addition to skeletal cells, immune cells have emerged as crucial regulators of bone remodeling (7). In the BM, mature B cells and plasma cells secrete considerable amounts of OPG through a process that depends on CD40–CD40L costimulation from T cells (8). Deficiencies in either B or T cells lead to impaired OPG production and are associated with reduced bone mass and osteoporosis in mice (8). Notably, conditional deletion of protein kinase C delta (PKC-δ, encoded by PRKCD) in B cells disrupts skeletal homeostasis by increasing the RANKL/OPG ratio, enhancing OC-mediated resorption, and compromising OB function, reinforcing the role of PKC-δ in bone–immune coupling (9). Interestingly, although B cells produce high levels of OPG on a per-cell basis, osteocytes and OBs remain the predominant *in vivo* sources that regulate bone mass, as demonstrated by the skeletal phenotype of mice with cell-specific OPG deletions in these populations (10–12). Disruptions in early B-cell development, such as impaired pre–B-cell receptor signaling, can lead to a concomitant reduction in both OB populations, ultimately resulting in generalized bone loss (12). This interdependence is further underscored by the other side of this cross-talk: Bone-resident B-cell development depends on IL-7 produced by OB-lineage cells (13).

Under pathologic or inflammatory conditions, B cells may undergo phenotypic reprogramming toward a pro-osteolytic profile. In diseases, such as human immunodeficiency virus (HIV) infection (14, 15), osteoporosis (16, 17), periodontal disease (18–20), and breast cancer–associated bone metastases (21), B cells upregulate RANKL, thereby promoting osteoclastogenesis. In HIV-1 transgenic mice, this shift is marked by reduced OPG and increased RANKL production by B cells along with monocytic alterations that expand the OC precursor pool in the BM (22). In HIV-positive individuals with severe CD4^+^ T-cell lymphopenia, the expansion of immature/transitional B cells, driven by elevated IL-7 levels, correlates strongly with an imbalance in the RANKL/OPG ratio in B cells and reduced bone mineral density (BMD; refs. 23, 24). Together, these findings emphasize the role of B cells in physiologic bone remodeling and their plasticity, as well as their ability to shift toward protective or pathogenic phenotypes depending on environmental cues. This duality holds significant implications for understanding their role in breast cancer–associated bone disease and identifying immune biomarkers predictive of metastatic risk.

In the metastatic setting associated with bone loss, dysregulated RANKL expression by the OB lineage and tumor-associated immune cells promotes excessive osteoclastogenesis and matrix degradation (21, 25, 26). This process releases growth factors that support tumor proliferation and survival in the BM, thereby establishing a self-perpetuating cycle of osteolytic metastases (26). Critically, these changes are preceded by immune and molecular alterations that precondition the BM for metastatic seeding (25, 27, 28). Indeed, we have previously shown that tumor-educated CD3^+^ T cells from the 4T1 model produce RANKL and trigger osteolysis, even in the absence of tumor cells (25). In BALB/c nude mice, the adoptive transfer of these T cells alone was sufficient to induce bone loss (25). Silencing RANKL completely abrogated this effect, confirming the critical role of tumor-primed T cells in premetastatic niche formation (25). More recently, we demonstrated that RANKL^+^ B cells act synergistically with tumor-specific T cells to promote bone metastasis (21). Additionally, dendritic cells carrying tumor antigens into the BM sustain the Th17 axis through IL-23 and undergo OC-like differentiation, further amplifying bone resorption (29). Conversely, inoculation with the *in situ* 67NR cell line, a nonmetastatic sibling of 4T1, elicited a protective immune profile characterized by CD8^+^ T cells producing IFN-γ and IL-10, leading to increased bone mass (30).

Here, we identified a subset of CD19^+^ B cells that produce OPG in response to 67NR tumor cues. These cells counteract the osteolytic effects of 4T1-primed T cells, thereby contributing to bone preservation. Notably, both the prometastatic RANKL^+^ and antimetastatic OPG^+^ expressing cells identified in murine models were also observed in lymphocytes infiltrating human triple-negative breast cancer (TNBC) primary tumors. This evolutionarily conserved immunologic axis may serve as a foundation for the development of prognostic biomarkers and targeted therapies aimed at mitigating bone metastases in breast cancer.

## Materials and Methods

Detailed experimental protocols were not deposited in external repositories. However, all procedures were conducted following standardized and reproducible workflows that are routinely adopted in our laboratory. These protocols have been extensively validated and are fully described in previous peer-reviewed publications by our group (21, 25, 29, 30).

### Ethical approval

All experiments involving animals and human specimens were conducted in accordance with institutional, national, and international guidelines and regulations. The animal protocols complied with the ARRIVE guidelines and were approved by the Ethical Committee for the Use of Experimental Animals of the Oswaldo Cruz Institute (license number L-002/2015). The retrospective study was conducted in accordance with the Declaration of Helsinki and approved by the Research Ethics Committee of the Federal University of Rio de Janeiro (protocol number 21941-913; CAE: 28003420.1.0000.5257).

### Animal experiments

Female BALB/cJ [Research Resource Identifier (RRID):IMSR_JAX:000651], BALB/c nude (RRID:IMSR_JAX:002019), and CBySm.n.Cg-Prkdc Scid J mice (referred to here as BALB/c SCID; RRID:IMSR_JAX:001802) aged 6 to 8 weeks and weighing approximately 20 to 25 g were used in all experiments and inoculated with 5 × 10^4^ 4T1 tumor cell line (RRID:CVCL_0125) or 67NR tumor cell line (RRID:CVCL_9717) in 100 μL into the fourth mammary fat pad, orthotopically or subcutaneously. The maximum diameter of the primary tumors was measured using digital calipers on predetermined days after injection. The 4T1 and 67NR tumor cell lines were cultured in DMEM (Gibco, RRID:SCR_021078) supplemented with 10% FBS (Gibco, RRID:SCR_022321) and 1% antibiotic-antimycotic solution (Gibco, RRID:SCR_019987). All cell lines used in this study were authenticated and regularly tested for *Mycoplasma* contamination using the MycoAlert Mycoplasma Detection Kit (Lonza, LT07 118) performed in-house. For preparation of soluble tumor antigen (sAg), animals were euthanized; tumors were dissected, resuspended in ice-cold PBS, filtered through a 40 μm cell strainer, subjected to five freeze-thaw cycles, boiled for 10 minutes, and centrifuged at 14,000 rpm for 30 minutes at 4°C. Animals were allocated to experimental groups based on predefined parameters to minimize biological variability, including age, body weight, and the use of a standardized tumor cell inoculum. All animals underwent an acclimatization period of approximately 1 week prior to experimental procedures to ensure physiologic stabilization and environmental adaptation. Occasional animal losses occurred during the course of the experiments due to causes unrelated to the experimental procedures. These events were rare and did not affect group allocation or data analysis. Group sizes were determined based on prior experience with similar experimental models and expected biological variability, rather than formal statistical power calculations. No formal power calculation was used; each experimental group included 4 to 5 animals, which has previously been sufficient to detect biologically relevant differences in tumor burden, bone metastases, lymphocyte adoptive transfers, and immune cell phenotypes.

### Clonogenic metastatic assay

Metastatic burden in lymph nodes (LN), BM, spleen, lungs, and liver was quantified using a clonogenic assay selective for 6-thioguanine–resistant tumor cells. Briefly, tissues were aseptically collected and processed to obtain single-cell suspensions. BM was mechanically dissociated and enzymatically digested using collagenase type I (1 mg/mL; Sigma-Aldrich, RRID:SCR_008988) and DNase I (100 μg/mL; Roche, cat. #10104159001) for 45 minutes at 37°C. Lungs and liver were digested using collagenase type IV (1 mg/mL; Sigma-Aldrich, RRID:SCR_013575 ) and DNase I (100 μg/mL; RRID:SCR_013575) under the same conditions. LNs and spleens were processed by gentle mechanical disruption only, without enzymatic digestion; no ACK lysis buffer was used for LNs. After filtration through a 70 μm mesh, cells from each tissue/mouse were plated in triplicate at limiting dilution in RPMI 1640 medium supplemented with 10% FBS and 6-thioguanine (4 μg/mL; Sigma-Aldrich, cat. #A4882, CAS 154427). Cultures were maintained for 10 to 14 days at 37°C with 5% CO_2_, after which colonies were fixed with 50% methanol and stained with 0.05% methylene blue. Only 6-thioguanine–resistant colonies were counted, and results were expressed as the number of metastatic clonogenic cells per 10^6^ plated cells.

### Conditioned medium preparation

CD3^+^ T cells and CD19^+^ B cells were isolated from the iliac BM of naive and 4T1 or 67NR tumor–bearing BALB/c J and BALB/c nude mice. Isolation was performed using magnetic labeling with PE-labeled rat anti-mouse CD3 monoclonal antibody (clone 145-2C11, BD Biosciences, RRID:AB_312685) and anti-PE microbeads (Miltenyi Biotech, RRID:AB_2783911) or PE-labeled rat anti-mouse CD19 monoclonal antibody (clone 1D3; BD Biosciences, RRID:AB_394659) and anti-PE microbeads (Miltenyi Biotech, RRID:AB_2783911). Isolated CD3^+^ T cells were then cocultured with CD11c^+^ splenic dendritic cells (DC) pulsed with 50 μg/mL sAg for 72 hours at 37°C, whereas CD19^+^ B cells were incubated with free sAg (50 μg/mL) for 24 hours at 37°C. After incubation, the supernatants were collected and referred to as conditioned medium (CM T 4T1 or CM B 67NR).

### *In vitro* assays for OC differentiation and activity

Iliac BM cells were cultured at a density of 1 × 10^5^ cells per well in 24-well plates using minimum essential medium (Life Technologies, RRID:SCR_022605) supplemented with 10% FBS (Gibco, RRID:SCR_022321). The cultures were maintained in the presence of: (i) M-CSF (10 ng/mL, PeproTech, RRID:SCR_013869); (ii) 50% CM, stimulated with sAg (50 μg/mL); or (iii) RANKL (10 ng/mL, PeproTech, RRID:SCR_013869). The cultures were incubated for 7 to 10 days at 37°C. Tartrate-resistant acid phosphatase (TRAP) activity in adherent OC cultures was assessed using the Leukocyte Acid Phosphatase Kit (Sigma-Aldrich, RRID:SCR_008988). TRAP-positive cells containing three or more nuclei were identified as OCs. To evaluate *in vitro* osteoclastic activity, cells were cultured as described above, and matrix resorption activity was detected according to the manufacturer’s protocol using the BD BioCoat Osteologic Bone Cell Culture System (BD Biosciences, RRID:SCR_018204).

### Cell sorting experiments

Primary 4T1 and 67NR tumors from BALB/c J mice were harvested in ice-cold PBS and enzymatically digested with 3.65 mg/mL collagenase (C9891, Sigma-Aldrich, RRID:SCR_008988), 12.5 mg/mL hyaluronidase (H3506, Sigma-Aldrich, RRID:SCR_008988), and 1 mg/mL DNase (Roche, RRID:SCR_013575), followed by mechanical dissociation using a 40 μm cell strainer (BD Biosciences). Draining LNs from female BALB/c J mice were collected in ice-cold PBS and mechanically dissociated using a 40 μm cell strainer without prior enzymatic digestion. The resulting single-cell suspensions were stained with AF488-conjugated rat anti-mouse CD45 (clone 30-F11, BD Biosciences, RRID:AB_2736991), PerCP Cy5.5-conjugated rat anti-mouse CD3 (clone 145-2C11, BD Biosciences, RRID:AB_312685), PECy7-conjugated rat anti-mouse CD4 (clone GK 1.5, BD Biosciences, RRID:AB_398644), APC-conjugated rat anti-mouse CD19 (clone 1D3, BD Biosciences, RRID:AB_394659), and viability stain (BD Horizon 780, RRID:AB_2869363). Prior to cell sorting, all samples were filtered through 40 μm membranes in PBS containing 1 mmol/L EDTA, 100 μg/mL DNase, and 2% FBS (Gibco). Data acquisition was performed on a FACSAria2 flow cytometer (BD Biosciences, RRID:SCR_018650) and analyzed using FlowJo software (Tree Star, RRID:SCR_008520).

### RNA extraction and OPG and RANKL gene expression analysis

RNA was extracted from sorted CD3^+^/CD4^+^ T cells and CD19^+^ B cells using the RNeasy Mini Kit (QIAGEN, RRID:SCR_015988) following the manufacturer’s instructions. Complementary DNA (cDNA) synthesis was performed using the GoScript Reverse Transcriptase Kit (Promega, RRID:SCR_020930) according to the manufacturer’s protocol. Quantitative reverse transcription PCR (qRT-PCR) was conducted using TaqMan assays (Thermo Fisher Scientific, RRID:SCR_002709) with the following primer probe sets from Applied Biosystems: *Tnfsf11* (Mm00441908_m1, Applied Biosystems, RRID: AB_2889614) and *Tnfrsf11b* (Mm00435452_m1, Applied Biosystems, RRID: AB_2837881). The relative mRNA expression levels were determined using the 2E-DDCt method.

### OPG and RANKL cytokine production

For OPG analysis, BM was harvested from the calvaria, humerus, iliac bone, femur, and tibia of BALB/cJ mice bearing 4T1 or 67NR tumors, as well as tumor-free controls. In separate experimental settings, BM was collected solely from the iliac bone of BALB/cJ and immunodeficient BALB/c nude or BALB/c SCID mice. BM tissues were subjected to enzymatic digestion using collagenase type I (1 mg/mL) and DNase I (100 μg/mL) at 37°C for 60 minutes, followed by mechanical dissociation to obtain single-cell suspensions. Following mechanical disruption, subpopulations of CD19^+^ B cells (total CD19^+^, CD19^+ ^IgM^+ ^IgD^+ ^CD138^+^, and CD19^+ ^IgM^+ ^IgD^+ ^CD138^−^) were isolated exclusively by sequential magnetic enrichment. Total CD19^+^ cells were first isolated by indirect magnetic labeling using PE-conjugated rat anti-mouse CD19 monoclonal antibody (clone 1D3; BD Biosciences), followed by anti-PE microbeads (Miltenyi Biotec, RRID: AB_2783911) and positive selection on LS columns. The purified CD19^+^ fraction was then subjected to additional rounds of magnetic separation to discriminate between plasma blast-like and mature-like B cell subpopulations. At each step, cells were relabeled, passed through microbeads, and collected as either CD138^+^ (retained) or CD138^−^ (flow-through) fractions. Total BM-derived cells were seeded at 10^7^ cells/mL, and sequentially isolated B cells, flow-sorted CD4^+^ T cells or CD19^+^ B cells from LNs and tumors [tumor-infiltrating lymphocytes (TIL)] were seeded at 10^6^ cells/mL in 24-well plates and cultured in the presence or absence of tumor-derived soluble antigens (sAg; 50 μg/mL). Supernatants were collected after 12 hours for OPG quantification (R&D Systems, cat. #DY459, RRID: AB_2802353) and after 72 hours for RANKL quantification (R&D Systems, cat. #DY462, RRID: AB_2802355), both performed by ELISA, as described in the figure legends.

### Adoptive transfer experiments

Iliac BM cells were isolated, and CD3^+^ T cells and CD19^+^ B cells were positively selected using magnetic beads coated with anti-mouse–specific antibodies (Miltenyi Biotec, cat. #130-094-973, RRID: AB_2736994 and cat. #130-121-301, RRID: AB_2872270, respectively). Isolated CD3^+^ T cells and CD19^+^ B cells (purity >94%) were adoptively transferred (1 × 10^6^ cells per mouse) intravenously into naive female BALB/c J, BALB/c nude, or BALB/c SCID mice (four or five mice per group) along with a single dose of 4T1 sAg (50 μg/mouse). In a separate experimental setup, BM-derived CD3^+^ T cells or CD19^+^ B cells from naive or 4T1 or 67NR tumor–bearing BALB/c J mice were injected intravenously, whereas 4T1 tumor cells were subcutaneously implanted into the mammary fat pad as an antigen source (five mice per group). Two weeks after the transfer, LN cells, splenocytes, and BM cells were stimulated *in vitro* with either sAg (50 μg/mL) or rat anti-mouse CD3 (1 μg/mL, cat. #553057, RRID: AB_394591). Nonstimulated cells from all groups served as controls. Flow cytometry was used to analyze the cells, and the supernatants were assessed for cytokine content via ELISA following established protocols.

### OPG silencing in CD19^+^ B cells from 67NR tumor–bearing mice and protein evaluation

To silence OPG expression in BM–derived CD19^+^ B cells from 67NR tumor–bearing BALB/c mice (day 11), cells were transfected with a specific murine short hairpin (sh) RNA (shRNA) plasmid targeting OPG [sc-40153-SH, Santa Cruz Biotechnology] or with a nontargeting control shRNA plasmid (sc-108060). Transfections were performed using the Amaxa Mouse B Cell Nucleofector Kit (Lonza, VPA-1010) according to the manufacturer’s instructions, with a final plasmid concentration of 3 μg per 2 × 10^6^ cells. Approximately 80% of viable cells were recovered 3 hours after transfection. These B cells were then adoptively transferred into either immunocompetent BALB/c or BALB/c SCID mice, together with subcutaneous inoculation of 4T1 tumor cells or intravenous administration of tumor sAg (50 μg/mouse), respectively. Transfection efficiency and OPG knockdown were confirmed by cell viability assessment and ELISA-based quantification of OPG in culture supernatants.

### Bone histomorphometry

Soft tissues were removed from the iliac bones of BALB/c J, BALB/c nude, and BALB/c SCID mice. The bones were fixed in 10% formalin, decalcified using a 20% EDTA solution (pH 5.5) for 2 weeks, and embedded in paraffin. EDTA decalcification was performed after formalin fixation to minimize artifacts or tissue alterations, ensuring better preservation for subsequent staining procedures. After 14 days, the decalcified bone became flexible and suitable for sectioning, following paraffin embedding. Although the mineral matrix was removed, the nonmineralized extracellular matrix remained intact, preserving the original shape of the bone tissue and allowing for the clear identification of spongy and cortical bone structures. Serial 5 μm paraffin-embedded sections were prepared and stained with hematoxylin and eosin (H&E) or TRAP (Sigma-Aldrich, RRID:SCR_008988) following standard protocols. H&E-stained slides were scanned using a digital slide scanner (ScanScope, Aperio, RRID:SCR_018457). Bone histomorphometric analyses were performed using semiautomated image analysis software (Motic). To assess trabecular volume [bone volume fraction (BV/TV)], a grid of 100 quadrants was used to standardize the analyzed areas across all samples. The total cortical and trabecular bone areas were considered 100%, and BV/TV measurements were performed on longitudinal sections from the dorsal, medial, and ventral regions of the iliac bones. Each animal contributed triplicate slides for the three regions, with at least five mice per group, to ensure normalized bone morphometric comparisons. Trabecular bone was distinguished from cortical bone based on its collagen framework, which was preserved despite EDTA demineralization. TRAP-positive OCs were quantified in the same sections and expressed as the number of cells per millimeter of bone length.

### Micro–computed tomography scanning, image reconstruction, and data processing and analysis

Soft tissues were excised from the iliac bones of the BALB/c J and BALB/c SCID mice. The bones were then fixed with 100% ethanol at 4°C. Micro-computed tomography (Micro-CT) scans were conducted using a Bruker Skyscan 1172 (Kontich). Each sample was wrapped in moist gauze, placed inside an Eppendorf tube, and scanned at 65 kV and 143 μA, with a rotation step of 0.59 degrees, 2 × 2 pixel binning, and an exposure time of 0.63 seconds per frame (three frames per projection). The resulting voxel size was 5 μm per pixel. To minimize low-energy X-ray interference, a 0.5 mm aluminum filter was employed. Micro-CT image reconstruction was performed using the NRecon software (version 1.7.5, Bruker, RRID:SCR_017377), applying a beam hardening correction of 40%, a ring artifact correction of eight, and no smoothing. The samples were consistently oriented to ensure reproducibility and uniformity across all scans. The volume of interest was defined using consistent anatomic landmarks in the distal femoral metaphysis to standardize the region analyzed across all animals. The trabecular bone was digitally segmented from the cortical bone using the CTAn software (version 1.20.3, Bruker, RRID:SCR_017372), and morphometric parameters were calculated following standardized guidelines for rodent bone microarchitecture analysis. The analyses included trabecular BV/TV, trabecular number, thickness, and separation. All reconstructions and analyses were conducted under blinded conditions. For each sample, 200 slices corresponding to a height of 1 mm were selected from the designated region. A task list in the CTAn facilitated the separation of cortical and trabecular areas, segmentation, and quantification of the analyzed parameters.

### Human specimens, IHC, and cell quantification

Paraffin-embedded sections of TNBC surgical tissue specimens were selected and identified using the RHC database (Cancer Hospital Registry and Surveillance System of the Clementino Fraga Filho University Hospital, Federal University of Rio de Janeiro) and included individuals with early-stage breast cancer who subsequently developed bone metastases as well as patients initially diagnosed with stage IV disease due to bone metastases (inclusion criteria). A matched control group comprising patients with the same prognostic factors who did not develop bone metastases and had a minimum follow-up of 5 years was also selected for comparison. This retrospective study was approved by the Federal University of Rio de Janeiro Research Ethics Committee under protocol number 21941-913 (CAE: 28003420.1.0000.5257). Written informed consent was obtained from all patients enrolled in this study, in accordance with institutional and national ethical guidelines. The tissue samples were fixed in 10% buffered formalin for 24 hours and embedded in paraffin. Histologic sections (5 μm thick) were obtained using a rotary microtome and mounted on positively charged glass slides (ImmunoSlide, EasyPath, RRID:SCR_031883). IHC staining was performed using the streptavidin–biotin–peroxidase complex technique. Paraffin-embedded tissue sections were deparaffinized in xylene and rehydrated in a graded ethanol series. Antigen retrieval was performed by heat-induced epitope retrieval using Leica Epitope Retrieval Solution (pH 6.0 and 9.0, Leica Biosystems, RRID:SCR_016559) according to the primary antibody by heating at 96°C for 40 minutes. Endogenous peroxidase activity was blocked using Leica Peroxidase Block (Leica Biosystems, RRID:SCR_016559) for 5 minutes, followed by nonspecific binding blockade using Leica Protein Block (Leica Biosystems, RRID:SCR_016559) for 5 minutes at room temperature. Sections were incubated with the following primary antibodies: anti-CD4 (clone SP35, Cell Marque, RRID:AB_2877883), anti-CD3 (clone MRQ-39, Cell Marque, RRID:AB_2880051), anti-CD20 (clone L26, Cell Marque, RRID:AB_2877881), anti-OPG (clone E-10, Santa Cruz Biotechnology, RRID:AB_627683), and anti-RANKL (clone G-1, Santa Cruz Biotechnology, RRID:AB_2189774) overnight at 4°C. After TBS washing, the sections were incubated for 30 minutes with a secondary biotinylated antibody (Leica Post Primary, Leica Biosystems, RRID:SCR_016559), followed by a 30-minute incubation with the Novolink Polymer Detection System (Leica Biosystems, RRID:SCR_016559). The chromogenic reaction was developed using diaminobenzidine (DAB), and the slides were counterstained with Harris hematoxylin. Quantitative analyses were performed using an optical microscope (OPTHD 3.7, Opticam Microscopy Technology) equipped with an image acquisition system. Three representative high-power fields (400× magnification) were selected for each section. Manual counting was performed independently by two blinded observers. Lymphocytes were first quantified based on morphologic criteria and marker-positive lymphocytic cells were identified and quantified. The results were expressed as the percentage of positively stained lymphocytes per field.

### Bioinformatic analysis

No custom code was developed for data analysis. For the prognostic significance of *TNFSF11* (RANKL) and *TNFRSF11B* (OPG) gene expression in association with immune cell infiltration in basal-like breast cancer (BRCA-Basal), Kaplan–Meier survival analyses were conducted using the publicly available TIMER 2.0 platform (http://timer.cistrome.org, RRID:SCR_016731), developed by the Dana-Farber Cancer Institute. The analyses focused on the impact of gene expression levels and the infiltration of CD4^+^ T cells and B cells on overall survival in patients with BRCA-Basal tumors. Survival curves were generated using the log-rank (Mantel–Cox) test, and the platform automatically computed hazard ratios (HR), 95% confidence intervals (CI), and *P* values, which were displayed in the resulting plots. No custom code was developed for data analysis.

### Statistical analysis

Quantitative data are presented as mean ± standard deviation (SD) or median with interquartile range (IQR), as indicated in each figure legend, based on the distribution and visualization method (e.g., bar graphs or box plots). Statistical comparisons between more than two groups were performed using one-way ANOVA followed by Tukey *post hoc* test for pairwise comparisons when data were normally distributed. For two-group comparisons, unpaired two-tailed Student *t* test, Mann–Whitney U test, and F test for variance homogeneity were employed depending on the sample characteristics. Given the small sample size (*n* = 5 per group in some cases), data normality was not formally assessed. Therefore, nonparametric tests were prioritized as the primary method of inference, with parametric tests used as confirmatory tools when applicable. All statistical analyses were performed using GraphPad Prism (version 8.0.1; GraphPad Software Inc.; RRID:SCR_002798), and a *P* value ≤ 0.05 was considered statistically significant.

### Resource identification and reproducibility (RRID compliance statement)

To promote transparency, rigor, and reproducibility in biomedical research, all key biological resources used in this study, including cell lines, antibodies, mouse strains, culture media, reagents, software, and instrumentation, have been cited along with their corresponding RRIDs. This practice is in accordance with the guidelines of the American Association for Cancer Research, the Resource Identification Initiative, and SciScore recommendations. A comprehensive Key Resources Table listing all reagents and tools, along with their respective RRIDs, is provided in the Supplementary Material (Supplementary Table S1).

## Results

### The 67NR nonmetastatic breast cancer is associated with increased OPG levels

**Figure 1.**
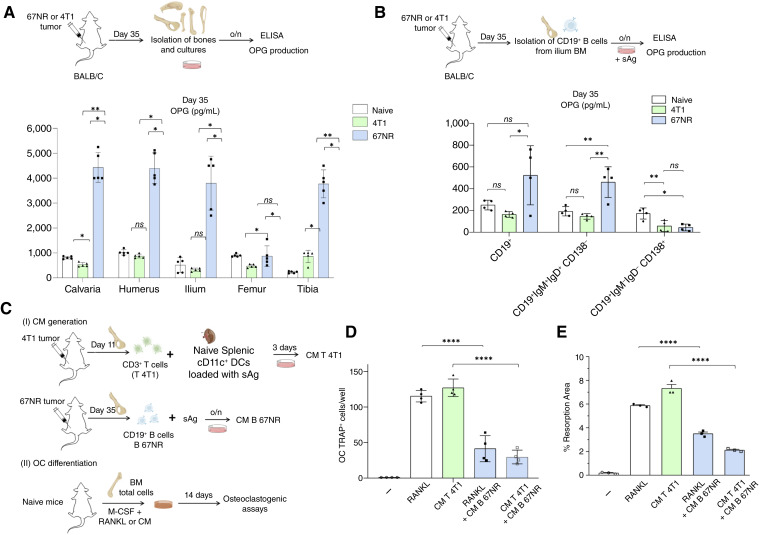
67NR-derived CD19^+ ^IgD^+ ^IgM^+ ^CD138^−^ B cells are the major source of OPG and inhibit *in vitro* osteoclastogenesis mediated by soluble factors derived from 4T1 tumor CD3^+^ T cells. **A,** Quantification of OPG secretion by total BM cells isolated from distinct skeletal sites: femur, tibia, vertebrae, humerus, and calvaria, of tumor-free, 67NR tumor–bearing, and 4T1 tumor–bearing mice at day 35 after implantation. A total of 10^7^ BM cells/mL were cultured *in vitro* for 12 hours, and OPG levels in supernatants were measured by ELISA. **B,** CD19^+^ B cells isolated from the iliac BM of naive, 67NR-bearing, or 4T1-bearing mice were sorted into CD19^+ ^IgD^+ ^IgM^+ ^CD138^−^ (mature cells) and CD19^+ ^IgD^− ^IgM^− ^CD138^+^ (plasm-like cells) subsets and were cultured *in vitro* (10^6^ cells/mL) for 12 hours, and OPG levels in supernatants were measured by ELISA. **C,** Experimental scheme: (i) 11 days after 4T1 tumor cell injection, CD3^+^ T cells were isolated from iliac BM and stimulated with naive splenic CD11c^+^ DCs pulsed with 4T1 sAg. Supernatants were harvested after 72 hours of incubation and referred to as CM T 4T1. (ii) Thirty-five days after 67NR tumor cell injection, CD19^+^ B cells were isolated from iliac BM and stimulated with 4T1 sAg. Supernatants were harvested after 24 hours of incubation and referred to as CM B 67NR. (iii) OC assays were performed with M-CSF plus either RANKL or CM T 4T1, added to naive total BM cells, in the absence or presence of CM B 67NR. **D,** Quantification of TRAP^+^ multinucleated OC cells was determined (magnification 40×). **E,** Functional assessment of OC-mediated resorption using the BD BioCoat Osteologic Bone Cell Culture System. All data are from three independent experiments and are presented as mean ± SD of 4 to 5 mice/group. Statistical comparisons between more than two groups were performed using one-way ANOVA followed by Tukey *post hoc* test for pairwise comparisons when data were normally distributed. Statistical significance was considered when *P* < 0.05; *, *P* < 0.05; **, *P* < 0.01; ****, *P* < 0.0001.

As we previously demonstrated, mice bearing 67NR nonmetastatic mammary tumors exhibited increased trabecular bone mass from day 11 to day 35 after implantation, characterized by decreased OC numbers, enhanced OB lineage cells, and an antiosteoclastogenic immune environment enriched in IFN-γ, IL-10, and regulatory CD8^+^ T cells (30). Conversely, mice bearing metastatic 4T1 tumors displayed rapid early bone loss linked to a pro-osteolytic immune response mediated by RANKL^+ ^CD3^+^ T cells and DCs with OC-like activity (25, 29). To further explore the molecular mechanisms underlying this differential modulation of bone remodeling, we investigated the local production of OPG in the distinct skeletal compartments of tumor-free animals and mice bearing either 67NR or 4T1 tumors (Fig. 1A). Total BM cells were isolated from distinct skeletal sites—calvaria, humerus, ilium, femur, and tibia—of tumor-free and 4T1- and 67NR-bearing BALB/cJ mice and cultured *in vitro* for 12 hours to assess local OPG secretion. Quantification by ELISA revealed a marked bone-wide increase in OPG production in 67NR-bearing animals compared with both control and 4T1 groups (Fig. 1A). This increase was particularly pronounced in trabecular-rich and highly vascularized bones such as the calvaria, ilium, and tibia, in which OPG levels were significantly elevated (*P* < 0.01–0.001) in 67NR-bearing mice. The humerus also exhibited a moderate but significant increase (*P* < 0.05), whereas the femoral shafts, characterized by dense cortical bone and lower hematopoietic activity (31), showed no difference between 67NR-bearing and tumor-free mice although OPG levels remained higher than in 4T1-bearing animals (Fig. 1A). These data demonstrate that the nonmetastatic 67NR tumors systemically enhance osteoprotective signaling throughout the skeleton, but the intensity of this response varies according to bone architecture and marrow composition. The preferential elevation of OPG in trabecular-rich bones suggests that these sites, which support active bone remodeling, may act as sentinel compartments for tumor-induced immunoregulatory responses that counteract osteolytic signaling associated with metastatic progression.

### CD19^+ ^IgD^+ ^IgM^+ ^CD138^−^ B cells from 67NR tumor–bearing animals are the main OPG-producing B cell subset in BM

To investigate whether tumor-associated modulation of the B cell compartment underlies the osteoanabolic phenotype observed in animals bearing 67NR tumors, we analyzed the production of OPG by CD19^+^ B cells isolated from the iliac BM (Fig. 1B). The iliac bone was used as the representative skeletal site for all subsequent experiments because it exhibited the most pronounced and consistent osteoimmune responses among the bones analyzed, providing an optimal microenvironment for evaluating immune-mediated regulation of bone remodeling (25). Total CD19^+^ B cells isolated from the iliac bones of tumor-free mice and animals bearing either 67NR or 4T1 tumors were cultured *in vitro* and stimulated with tumor-derived sAg (Fig. 1B). ELISA quantification revealed that CD19^+^ B cells from 67NR tumor–bearing animals secreted higher amounts of OPG than those from 4T1-bearing mice, with this difference reaching statistical significance with *P* = 0.0272 (Fig. 1B). A trend toward increased OPG secretion was also observed when comparing 67NR-bearing to tumor-free animals (*P* = 0.0892), whereas no difference was detected between them and the 4T1 groups with *P* = 0.7326 (Fig. 1B). As expected, B cells from naive mice produced baseline levels of OPG, consistent with their physiologic contribution to bone homeostasis (Fig. 1B). To further delineate which specific B cell subsets contributed most significantly to OPG production, we phenotypically stratified and sorted CD19^+^ B cells into two major subpopulations based on their surface marker expression: mature recirculating (CD19^+ ^IgD^+ ^IgM^+ ^CD138^−^) and plasma blast-like (CD19^+ ^IgD^− ^IgM^− ^CD138^+^) subsets (Fig. 1B). Among these, CD19^+ ^IgD^+ ^IgM^+ ^CD138^−^ mature-like cells emerged as the predominant OPG producers in 67NR tumors (Fig. 1B), as compared with their counterparts from both naive and 4T1 groups (Fig. 1B). These findings support the notion that the BM B cell compartment is functionally modulated in response to nonmetastatic tumor burden, with specific enrichment of an OPG-producing phenotype among IgD^+ ^IgM^+ ^CD138^−^ B cells, in accordance with previous findings (8).

### Anti-osteoclastogenic soluble factors from 67NR-primed CD19^+^ B cells counteract 4T1 tumor-specific CD3^+^ T cell–induced osteoclastogenesis

We next assessed whether OPG-producing CD19^+^ B cells could functionally suppress osteoclastogenesis driven by RANKL^+^ CD3^+^ T cells in 4T1-bearing animals. To this end, we performed *in vitro* osteoclastogenic assays in which naive OC precursors were stimulated with M-CSF and RANKL or CM derived from CD3^+^ T cells isolated from the BM of 4T1 tumor-bearing mice (CM T 4T1; Fig. 1C). As expected, T cell–derived CM induced robust osteoclastogenesis, as measured by the number of TRAP^+^ multinucleated OCs (Fig. 1D). However, when CM from CD19^+^ B cells isolated from 67NR-bearing mice (CM B 67NR) was added to the culture, marked inhibition of OC formation was observed (Fig. 1D). Notably, this suppressive effect was evident both in cultures in which osteoclastogenesis was directly induced by recombinant RANKL and in those stimulated by CM from 4T1 tumor-specific CD3^+^ T cells (Fig. 1D). These findings indicate that CM B 67NR exerts a broad inhibitory effect on osteoclastogenesis, regardless of whether it is induced by defined or tumor-driven stimuli (Fig. 1D). To evaluate whether this suppression extended to OC functional bone resorptive activity, synthetic bone matrix degradation assays were performed under the same conditions (Fig. 1E). The copresence of CM B 67NR significantly reduced the resorptive area induced by CM T 4T1, indicating the impaired functional capacity of OCs (Fig. 1E). Together, these results indicate that CD19^+^ B cells primed in the 67NR tumor context acquire regulatory functions capable of counteracting pro-osteoclastogenic signals mediated by metastatic tumor-specific CD3^+^ T cells previously shown to be RANKL-dependent through OPG production (25).

### Adoptive transfer of CD19^+^ B cells from 67NR tumor–bearing mice reduces RANKL production by 4T1-specific CD3^+^ T cells, restores trabecular bone mass *in vivo*, and inhibits 4T1 metastatic dissemination *in vivo*

**Figure 2.**
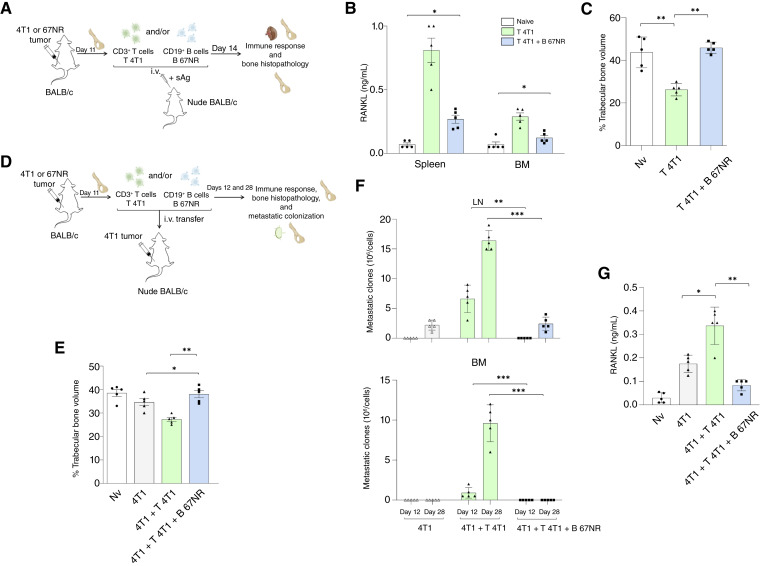
Adoptive transfer of 67NR-derived CD19^+^ B cells reduces RANKL production by 4T1 tumor–specific CD3^+^ T cells, inhibits bone resorption, and prevents bone metastases in BALB/c nude recipient mice. **A,** Experimental scheme: CD3^+^ T cells were magnetically isolated from the iliac BM of 4T1 tumor-bearing BALB/c mice on day 11 after implantation, and CD19^+^ B cells were isolated from the BM of 67NR tumor–bearing mice at the same time point. The cells were transferred intravenously into BALB/c nude mice either alone (T 4T1) or in combination (T 4T1 + B 67NR), followed by *in vivo* restimulation with 4T1 tumor sAgs. Naive mice were used as controls (Nv). **B,** RANKL levels were quantified by ELISA in culture supernatants of total spleen and BM cell suspensions obtained from recipient mice 14 days after transfer, following *in vitro* restimulation with 4T1 sAg. Each value reflects total RANKL concentration normalized to cell number (1 × 10^6^ cells/mL), representing the overall functional output of the transferred T cell populations within the respective tissue compartments. T 4T1 means mice that received only 4T1+ CD3^+^ T cells with sAg, and T 4T1 + B 67NR means mice that received both 4T1+ CD3^+^ T cells and 67NR+ CD19^+^ B cells with sAg. Naive mice were used as experimental controls (Nv). **C,** Histomorphometric analysis of iliac bones was performed 14 days after cell transference. Sagittal sections from demineralized iliac bones were made following conventional methods and stained with H&E. All microscope slides were scanned with a ScanScope GL equipped with a 40× objective. Trabecular bone volume was expressed as a percentage of total tissue volume (% BV/TV). **D,** Experimental design for the metastatic setting: BALB/c nude mice were injected orthotopically with 4T1 tumor cells and subsequently received the adoptive transfer of CD3^+^ T cells from 4T1-bearing mice with or without CD19^+^ B cells from 67NR-bearing donors on day 11 after tumor injection. **E,** Histomorphometric analysis of iliac bones was performed on days 12 and 28 after cell transference, as described in **C**. **F,** Metastatic colonization was assessed on days 12 and 28 by clonogenic metastatic assays in draining LNs and BM. **G,** RANKL quantification by ELISA in culture supernatants of *in vitro* restimulated LN-derived cells with sAg, collected from the same experimental groups at endpoint. All data represent mean ± SD of five mice per group and are representative of at least two independent experiments. Statistical comparisons between more than two groups were performed using one-way ANOVA followed by Tukey *post hoc* test for pairwise comparisons when data were normally distributed. Statistical significance was considered when *P* < 0.05; *, *P* < 0.05; **, *P* < 0.01; ***, *P* < 0.001.

We previously demonstrated that tumor progression and metastatic spread in athymic BALB/c nude mice require the adoptive transfer of CD3^+^ T cells derived from 4T1 tumor-bearing donors (25). To investigate the *in vivo* impact of 67NR-primed CD19^+^ B cells, we performed adoptive transfer experiments in immunodeficient BALB/c nude mice to assess RANKL production and bone remodeling parameters. The time point chosen for B-cell isolation (day 11 after tumor inoculation) corresponds to the peak of OPG secretion by CD19^+^ B cells, which presents the kinetic profile of OPG production throughout 67NR tumor progression (Supplementary Fig. S1). Recipient mice received CD3^+^ T cells and/or CD19^+^ B cells isolated from the iliac BM of the 4T1- or 67NR-bearing donor mice, respectively (Fig. 2A). Fourteen days after cell transfer, splenocytes and BM cells were harvested and restimulated *in vitro* with 4T1 sAg (Fig. 2A). RANKL levels in the culture supernatants were quantified by ELISA. As expected, 4T1-primed CD3^+^ T cells produced high levels of RANKL upon antigenic stimulation (Fig. 2B). Notably, cotransfer of 67NR–derived CD19^+^ B cells significantly reduced RANKL production by 4T1 T cells from both spleen and BM cultures (Fig. 2B). In parallel, histomorphometric analyses of the iliac trabecular bone revealed that cotransfer of 67NR–derived CD19^+^ B cells significantly restored the bone mass loss induced by 4T1 tumor–specific CD3^+^ T cells (Fig. 2C). These findings indicate that CD19^+^ B cells primed in the context of nonmetastatic 67NR tumors retain regulatory properties *in vivo*, effectively counteracting the osteolytic activity driven by 4T1 pro-resorptive CD3^+^ T cells. We next sought to evaluate whether 67NR-primed CD19^+^ B cells could interfere with the broader protumoral program induced by 4T1-specific CD3^+^ T cells. For this purpose, 4T1-primed CD3^+^ T cells were transferred alone or in combination with 67NR-primed CD19^+^ B cells into 4T1 tumor-implanted nude recipients (Fig. 2D). On day 28 after tumor implantation and adoptive transfer, histomorphometric analysis confirmed that 4T1 tumors combined with 4T1-primed CD3^+^ T cells led to pronounced bone loss *in vivo* (25), a phenotype that was substantially reversed upon cotransfer of 67NR-primed CD19^+^ B cells (Fig. 2E). Moreover, 67NR-primed CD19^+^ B cells inhibited metastatic colonization of both draining LNs and the BM, as assessed on days 12 and 28 after transfer (Fig. 2F). To investigate whether these protective effects could be mechanistically linked to the modulation of RANKL expression, we measured the cytokine levels in *in vitro* sAg-stimulated BM cells (Fig. 2G). As expected, 4T1 tumor-bearing nude mice that received 4T1-primed CD3^+^ T cells produced high amounts of RANKL in response to sAg stimulation. Conversely, the cotransfer of 67NR-primed CD19^+^ B cells significantly reduced RANKL production by CD3^+^ T cells (Fig. 2G). Collectively, these results demonstrate that 67NR–derived CD19^+^ B cells inhibited multiple hallmarks of bone metastatic disease, including T cell–mediated RANKL production, tumor-induced bone resorption, and colonization of skeletal tissues, reinforcing their role as negative regulators of bone metastatic disease *in vivo*.

### 67NR-primed CD19^+^ B cells suppress 4T1 primary tumor growth, osteolytic disease, and metastatic dissemination in immunocompetent BALB/c mice at early stages of tumor progression

**Figure 3.**
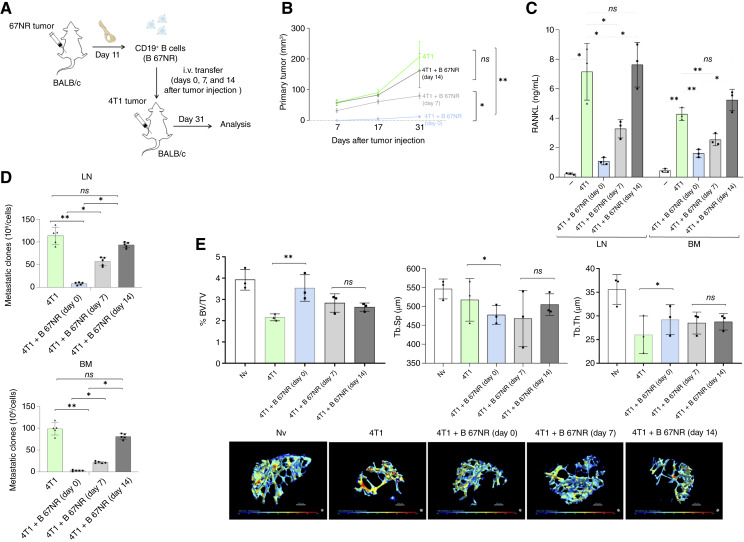
Early-stage acquisition of a regulatory phenotype by CD19^+^ B cells from 67NR tumor–bearing mice is required for optimal suppression of 4T1 tumor progression, RANKL production, bone loss, and metastasis. **A,** Experimental scheme: CD19^+^ B cells were isolated from the iliac BM of 67NR tumor–bearing BALB/c mice on day 11 after tumor implantation. These cells were transferred intravenously into BALB/c mice injected with 4T1 tumor cells at different time points: day 0, day 7, or day 14 after tumor injection. Mice were euthanized on day 31 for analysis. **B,** Primary tumor growth was monitored over time, and tumor volume was calculated on the indicated days. **C,** On day 31, RANKL production was measured by ELISA in supernatants from splenic and BM cell cultures restimulated *in vitro* with sAg. **D,** Metastatic burden was evaluated by clonogenic assays in draining LNs and BM. **E,** Micro-CT analysis of iliac bones was performed to assess structural parameters of trabecular bone, including BV/TV, trabecular separation (Tb.Sp), and trabecular thickness (Tb.Th). Representative 3D reconstructions of trabecular bone are shown below the graphs. Data are shown as mean ± SD (*n* = 4–5 mice per group), representative of at least two independent experiments. Statistical comparisons between more than two groups were performed using one-way ANOVA followed by Tukey *post hoc* test for pairwise comparisons when data were normally distributed. Statistical significance was considered when *P* < 0.05; *, *P* < 0.05; **, *P* < 0.01; Nv, naive control.

To assess whether 67NR-primed CD19^+^ B cells retain their immunomodulatory properties in an immunocompetent host, opening the possibility of their immunotherapeutic role, we adoptively transferred CD19^+^ B cells from the BM of 67NR tumor–bearing donors into BALB/c mice orthotopically implanted with 4T1 tumor cells (Supplementary Fig. S2A). On day 28 after tumor injection, we assessed immune responses, bone pathology, and metastatic dissemination. Mice receiving adoptive transfer of 67NR-derived CD19^+^ B cells exhibited reduced RANKL production by LN and BM immune cells upon 4T1 sAg restimulation compared with those receiving tumor alone or tumor plus naive B cells (Supplementary Fig. S2B). This was accompanied by partial inhibition of trabecular bone volume loss (Supplementary Fig. S2C), decreased primary tumor growth (Supplementary Fig. S1D), and reduced metastatic burden in both the LNs and BM (Supplementary Fig. S1E). We next sought to determine whether the timing of adoptive transfer affects its regulatory efficacy *in vivo*, as would be necessary for therapeutic use. To this end, B cells from 67NR were adoptively transferred into BALB/c mice receiving 4T1 tumor cells at different time points: on the day of tumor cell injection or on day 7 or 14 after 4T1 inoculation. All the mice were euthanized on day 31 for analysis (Fig. 3A). Tumor growth analysis revealed that early transfer of B 67NR cells at day 0 significantly suppressed primary tumor expansion, reduced RANKL production by immune cells, and preserved bone integrity compared with later time points (Fig. 3B–E). Delayed transfer at day 7 partially inhibited tumor growth and bone metastasis, partially modulating immune parameters and bone morphology. However, day 14 transfer had no effect on any of the parameters analyzed (Fig. 3B–E). Notably, the protective effect was restricted to the bone compartment, as confirmed by clonogenic assays showing colonization in the lung and liver (Supplementary Fig. S3A), indicating that OPG^+^ B cells primarily exert a local regulatory function within the osteoimmune niche rather than a systemic anti-metastatic effect. Moreover, these findings highlight that the timing of B cell–based transference is critical for its efficacy. Only the early-stage transfer of CD19^+^ B cells from 67NR tumor–bearing donors completely suppressed RANKL-mediated osteolytic activity, limited tumor growth, and impaired metastatic dissemination. In contrast, once osteolytic disease was established by day 7, only partial inhibition was observed, suggesting that B cells could be harnessed as an immunotherapeutic approach to mitigate tumor progression when applied during early disease stages.

### CD3^+^ T cells from 67NR mice bearing tumors are not required for the full inhibitory activity of CD19^+^ B cells *in vivo*

**Figure 4.**
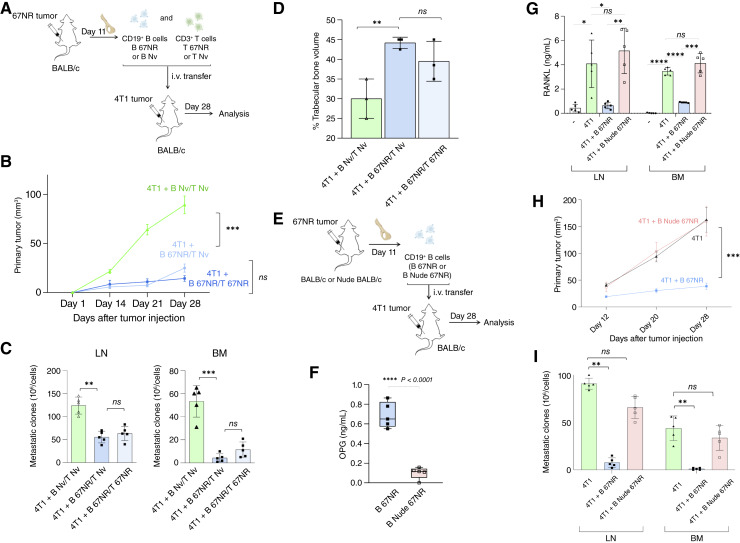
CD3^+^ T cells are dispensable for the effector function but required for the acquisition of the regulatory phenotype of CD19^+^ B cells *in vivo*. **A,** Experimental scheme: CD19^+^ B cells and CD3^+^ T cells were isolated from the iliac BM of naive or 67NR tumor–bearing BALB/c mice at day 11 after implantation and transferred intravenously into immunocompetent BALB/c recipients implanted with 4T1 tumor cells. Animals were grouped as follows: B Nv/T Nv, B 67NR/T Nv, and B 67NR/T 67NR (B and T cells from 67NR-bearing mice). **B,** The maximum area of primary tumors was determined using digital caliper measures. Tumor growth was monitored longitudinally and is shown as mean tumor volume (mm^3^) per group at regular intervals. **C,** On day 28, clonogenic assays were performed in draining LNs and BM. **D,** Histomorphometric analysis of sagittal iliac bone sections was performed to assess trabecular bone volume, expressed as a percentage of total tissue volume. **E,** Experimental scheme in which CD19^+^ B cells were isolated from the iliac BM of B 67NR or B Nude 67NR mice and transferred into 4T1 tumor–bearing immunocompetent BALB/c recipients. **F,** Quantification of OPG production by *in vitro* cultured CD19^+^ B cells isolated from B 67NR and B Nude 67NR donors. OPG levels were measured in culture supernatants by ELISA after 24 hours. **G,** RANKL levels were measured by ELISA in the supernatants of LN and BM cells restimulated with 4T1 sAg (sAg) *in vitro*. **H,** Primary tumor growth kinetics over time in mice receiving B 67NR or B Nude 67NR cells, compared with tumor-only controls. **I,** Clonogenic assays quantifying metastatic colonies in LNs and BM on day 28 after transfer. Data are expressed as mean ± SD from two independent experiments with *n* = 5 mice per group. Statistical comparisons between more than two groups were performed using one-way ANOVA followed by Tukey *post hoc* test for pairwise comparisons when data were normally distributed. Statistical significance was considered when *P* < 0.05; *, *P* < 0.05; **, *P* < 0.01; ***, *P* < 0.001.

To investigate whether the regulatory function of CD19^+^ B cells primed in 67NR tumors depends on CD3^+^ T cell activity, we performed adoptive transfer experiments using immunocompetent BALB/c mice (Fig. 4A). CD19^+^ B and CD3^+^ T cells were isolated from the iliac BM of either 67NR tumor–bearing or naive donors and transferred in different combinations into BALB/c mice orthotopically implanted with 4T1 metastatic tumor cells. On day 28 after implantation, we analyzed tumor growth, bone pathology, and metastatic dissemination (Fig. 4A). As expected, mice that received naive B and T cells (B Nv/T Nv) showed progressive 4T1 tumor expansion (Fig. 4B). In contrast, a significant reduction in primary tumor volume was observed in mice receiving cotransfer of 67NR-primed CD19^+^ B cells with naive T cells (B 67NR/T Nv), or both CD19^+^ B and CD3^+^ T cells from 67NR-bearing donors (B 67NR/T 67NR; Fig. 4B). Consistent with these findings, clonogenic assays on day 28 confirmed reduced metastatic colonization in the LNs and BM of mice that received 67NR-derived CD19^+^ B cells (Fig. 4C). Notably, this effect occurred regardless of whether B 67NR cells were transferred alongside CD3^+^ T cells from 67NR-bearing or naive animals, in contrast to the enhanced metastatic burden observed in the B Nv/T Nv group (Fig. 4C). Finally, histomorphometric analysis of the iliac trabecular bone showed that B 67NR cells, transferred either alone (B 67NR/T Nv) or together with T 67NR cells, significantly preserved bone mass compared with the group receiving B Nv/T Nv (Fig. 4D). Together, these findings indicate that 67NR-primed CD19^+^ B cells exert antiosteolytic effects independently of CD3^+^ T cells.

### CD3^+^ T cells are required to license the regulatory OPG-producing phenotype of 67NR-primed CD19^+^ B cells

To determine whether CD3^+^ T cells are required for the acquisition of an OPG-producing regulatory phenotype, we first quantified OPG secretion by CD19^+^ B cells isolated from the iliac BM of 67NR tumor–bearing wild-type BALB/c (B 67NR) and T cell–deficient nude BALB/c (B Nude 67NR) mice 11 days after tumor implantation (Fig. 4E and F). B cells derived from nude donors exhibited significantly lower OPG production than their wild-type counterparts (Fig. 4F), suggesting that priming by CD3^+^ T cells is necessary to induce the OPG-expressing phenotype. To test their functional relevance, these B cells were adoptively transferred into immunocompetent BALB/c recipients bearing 4T1 tumors (Fig. 4E). As previously shown, the cotransfer of wild-type B 67NR cells significantly reduced RANKL secretion in sAg-restimulated LN and BM cultures from 4T1-bearing recipients (Fig. 4G). In sharp contrast, B cells derived from nude 67NR donors failed to reduce RANKL production, showing cytokine levels comparable with those observed in 4T1 tumor–only mice (Fig. 4G). This inability to restrain RANKL expression was associated with exacerbated disease progression, as mice receiving B Nude 67NR cells developed tumors comparable in volume to those observed in tumor-only controls, whereas recipients of wild-type B 67NR cells displayed reduced tumor growth (Fig. 4H). Similarly, the protective effect of B 67NR cells against metastatic dissemination to LNs and the BM was completely lost in the absence of prior T cell priming, as demonstrated by the significantly higher number of metastatic colonies in mice receiving B Nude 67NR cells (Fig. 4I). Taken together, these results demonstrate that the acquisition of the OPG-producing regulatory phenotype by B cells requires T cell–mediated licensing during the initial tumor-priming phase.

### Silencing of OPG in 67NR-primed CD19^+^ B cells abrogates their regulatory activity and restores osteolytic and metastatic phenotypes in 4T1 tumor-bearing mice

**Figure 5.**
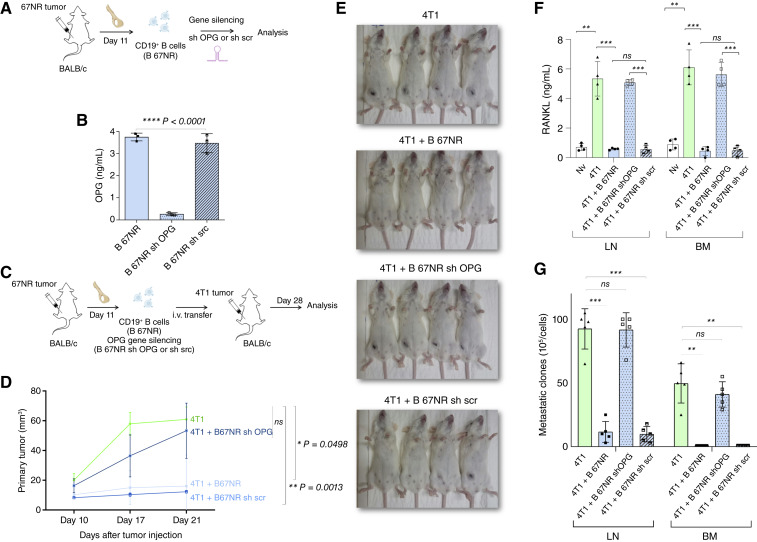
Silencing of OPG in 67NR-primed CD19^+^ B cells abrogates their regulatory activity and restores 4T1 osteolytic and metastatic phenotypes *in vivo*. **A,** Experimental scheme: CD19^+^ B cells were isolated from the iliac BM of BALB/c mice bearing 67NR tumors on day 11 after implantation. Cells were subjected to gene silencing using lentiviral vectors carrying shRNA targeting *Tnfrsf11b* (OPG; B 67NR sh OPG) or a nontargeting scrambled control (B 67NR sh scr). Naive mice were used as experimental controls (Nv). **B,** OPG levels in culture supernatants from CD19^+^ B cells were assessed by ELISA. **C,** Experimental setup for *in vivo* analysis: OPG-silenced (sh OPG) or control-transduced (sh scr) CD19^+^ B cells were adoptively transferred together with CD3^+^ T cells from 4T1 tumor-bearing mice into immunocompetent BALB/c mice implanted with 1 × 10^5^ 4T1 tumor cells. **D,** Primary tumor growth was monitored over time. **E,** Representative images of tumor-bearing mice after adoptive B cell transfer. Photographs were taken at the experimental endpoint to illustrate macroscopic differences in tumor burden among groups. **F,** Quantification of RANKL levels by ELISA in supernatants of *in vitro* restimulated LNs and BM cells. **G,** Metastatic burden in draining LNs and BM was assessed by clonogenic assays. Data are presented as mean ± SD from two independent experiments (*n* = 5 mice/group). Statistical comparisons between more than two groups were performed using one-way ANOVA followed by Tukey *post hoc* test for pairwise comparisons when data were normally distributed. Statistical significance was considered when *P* < 0.05; *, *P* < 0.05; **, *P* < 0.01; ***, *P* < 0.001.

To investigate whether OPG expression is essential for the regulatory function of CD19^+^ B cells primed in 67NR tumors, we performed gene silencing experiments targeting *Tnfrsf11b* (OPG) using shRNA (Fig. 5A). Flow cytometric analysis confirmed the high purity of the CD19^+^ population after isolation (>96%) and the effective depletion of contaminating CD3^+^ T cells (Supplementary Fig. S4). ELISA measurements showed that OPG protein levels were significantly reduced in OPG-silenced B cells compared with those in controls, confirming successful gene silencing (Fig. 5B). To evaluate the functional impact of OPG depletion *in vivo*, CD19^+^ B cells transfected with sh OPG or sh scrambled (sh scr) were cotransferred with 4T1-derived CD3^+^ T cells into immunocompetent BALB/c recipients bearing orthotopic 4T1 tumors (Fig. 5C). On day 28 after injection, we assessed disease burden and immune response. Strikingly, longitudinal tumor growth analysis demonstrated that OPG silencing in B 67NR cells resulted in loss of tumor control (Fig. 5D and E). Mice receiving cells of 67NR tumor–bearing donors that had been transduced with OPG-targeting shRNA (B 67NR sh OPG) exhibited tumor progression comparable with that of the 4T1 group, whereas those receiving B 67NR or 67NR tumor–bearing donors that had been transduced with a scrambled control (B 67NR sh scr) maintained reduced tumor burden throughout the observation period (Fig. 5D and E). Moreover, cotransfer of scrambled shRNA-transfected 67NR B cells suppressed RANKL production by 4T1-derived T cells in LNs and BM (Fig. 5F). In contrast, silencing of OPG expression in these B cells abrogated their suppressive effect, indicating that OPG is essential for B cell–mediated modulation of pro-osteolytic T cell responses (Fig. 5F). In line with these findings, clonogenic assays revealed that B 67NR sh OPG cells failed to prevent metastatic dissemination in both LNs and the BM (Fig. 5G). These results confirmed that OPG is indeed a B cell mediator that acts in the bone microenvironment.

### OPG produced by CD19^+^ B cells from 67NR tumor–bearing mice is required to restrain 4T1-specific T cell–derived RANKL production and bone loss

**Figure 6.**
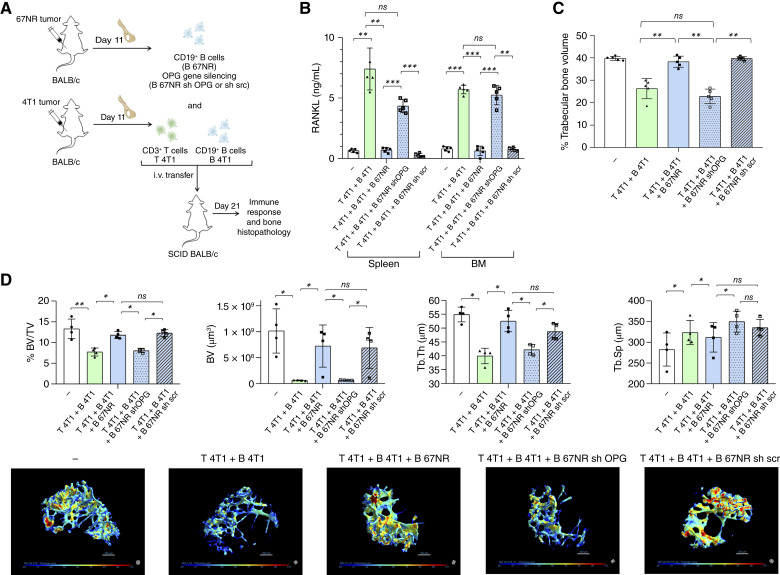
OPG produced by 67NR-primed CD19^+^ B cells is required to suppress RANKL production and protect bone structure *in vivo*. **A,** Experimental design: CD3^+^ T cells and CD19^+^ B cells were isolated from the iliac BM of 4T1 tumor–bearing BALB/c mice on day 11 after implantation. In parallel, CD19^+^ B cells from 67NR tumor–bearing BALB/c mice were transduced with sh OPG or sh scr. All cell types were adoptively cotransferred into BALB/c SCID recipient mice. On day 21, after transfer, animals were euthanized and analyzed. Naive mice were used as experimental controls (Nv). **B,** RANKL levels were quantified by ELISA in supernatants of spleen and BM cells restimulated *in vitro* with 4T1 sAg. **C,** Histomorphometric analysis was performed on the iliac bones from mice of the different groups. Sagittal sections from demineralized iliac bones were made following conventional methods and stained with H&E. All microscope slides were scanned with a ScanScope GL equipped with a 40× objective. Trabecular bone volume was expressed as a percentage of total tissue volume. **D,** Micro-CT analysis of iliac bones assessed structural parameters, including BV/TV, total trabecular volume (BV), trabecular thickness (Tb.Th), and trabecular separation (Tb.Sp), alongside representative 3D reconstructions. Data are shown as mean ± SD from two independent experiments with *n* = 5 mice per group. Statistical comparisons between more than two groups were performed using one-way ANOVA followed by Tukey *post hoc* test for pairwise comparisons when data were normally distributed. Statistical significance was considered when *P* < 0.05; *, *P* < 0.05; **, *P* < 0.01; ***, *P* < 0.001.

To directly determine whether the protective function of CD19^+^ B cells primed in the 67NR tumor context depends on their capacity to produce OPG, we employed a stringent experimental model using BALB/c SCID mice that are genetically deficient in both T and B lymphocytes. This approach ensures that all observed immunologic effects arise exclusively from adoptively transferred lymphocyte populations, thereby allowing precise dissection of B cell–intrinsic mechanisms (Fig. 6A). Importantly, all recipient mice in this experimental set received both CD3^+^ T cells and CD19^+^ B cells isolated from 4T1 tumor–bearing mice, which are known to drive robust pro-osteolytic responses. The experimental variable was the cotransfer of CD19^+^ B cells from B 67NR sh OPG or B 67NR sh scr. Twenty-one days after transfer, total spleen and BM cells from each group were cultured *in vitro* and restimulated with 4T1 tumor–derived sAg (Fig. 6). Soluble RANKL levels in the culture supernatants were quantified by ELISA as a functional readout of antigen-specific osteoclastogenic activity (Fig. 6A and B). Mice receiving only 4T1-derived CD3^+^ T and CD19^+^ B cells released high amounts of RANKL upon sAg stimulation (Fig. 6B). This response was markedly reduced by the cotransfer of wild-type B 67NR or B 67NR sh scr cells, but not by B 67NR sh OPG cells, indicating that OPG expression in 67NR B cells is required to restrain the antigen-dependent secretion of RANKL by 4T1-specific T cells rather than to directly regulate RANKL gene expression (Fig. 6B). Histomorphometric analysis of the iliac trabecular bone revealed that bone volume was preserved in animals receiving B 67NR or B 67NR sh scr cells but not in those receiving B 67NR sh OPG (Fig. 6C). Micro-CT confirmed these findings, showing decreased trabecular volume, thickness, and BV/TV in the absence of B cell–derived OPG [Fig. 6D (top)]. Three-dimensional reconstructions visually supported this interpretation, revealing extensive trabecular degradation in the B 67NR sh OPG group [Fig. 6D (bottom)]. Altogether, these data indicate that B cell–derived OPG is essential for limiting antigen-driven RANKL secretion by 4T1-specific T cells and for counterbalancing pro-osteolytic immune signaling, thereby preserving bone architecture. The use of SCID hosts cotransferred with pro-osteolytic 4T1-derived lymphocytes across all experimental groups reinforces that the protective effect arises specifically from the OPG-producing function of the transferred 67NR B cells.

### Tumor-infiltrating CD3^+ ^CD4^+^ T cells and CD19^+^ B cells from 4T1 and 67NR tumor–bearing mice exhibit distinct profiles associated with the RANKL–OPG axis

**Figure 7.**
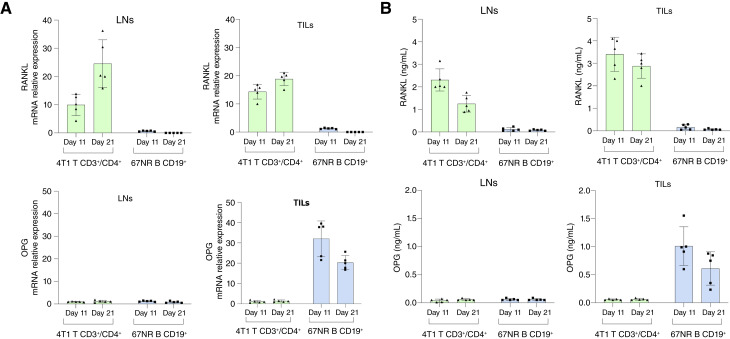
Tumor-infiltrating CD4^+^ T and CD19^+^ B cells from 4T1 and 67NR tumor–bearing mice exhibit distinct RANKL and OPG profiles. **A,** mRNA expression of *Tnfsf11* (RANKL) and *Tnfrsf11b* (OPG) in CD4^+^ T and CD19^+^ B cells isolated from tumors and LNs of mice bearing 4T1 or 67NR tumors, evaluated by qRT-PCR. Results are normalized and expressed as fold change relative to naive controls. **B,** Quantification of secreted RANKL and OPG in culture supernatants of CD3^+ ^CD4^+^ T cells (left) and CD19^+^ B cells (right) restimulated with 4T1 tumor sAg by ELISA. Data are presented as mean ± SD and are representative of two independent experiments (*n* = 5 mice per group). Statistical significance was considered at *P* < 0.05. Statistical comparisons between more than two groups were performed using one-way ANOVA followed by Tukey *post hoc* test for pairwise comparisons when data were normally distributed.

Given that human breast cancer samples available for translational studies are typically obtained from surgical specimens of the primary tumor, we sought to investigate whether the immune phenotypes previously identified in the lymphoid compartments of 4T1- and 67NR-bearing mice were also present within the tumor microenvironment. To this end, CD3^+ ^CD4^+^ T cells and CD19^+^ B cells infiltrating primary tumors (TILs) and draining LNs were sorted on days 11 and 21 after tumor implantation (Supplementary Fig. S5A and S5B) and cultured *in vitro* in the presence of 4T1 sAg (Fig. 7A). CD3^+ ^CD4^+^ T cells derived from 4T1-bearing mice exhibited marked upregulation of *Tnfsf11* (RANKL) mRNA in both LNs and TILs, which increased over time (day 11–21), supporting the acquisition of a pro-osteolytic transcriptional program (Fig. 7A). In contrast, CD19^+^ B cells from 67NR-bearing mice showed negligible expression of *Tnfsf11* at both time points and in tissue compartments (Fig. 7A). Notably, *Tnfrsf11b* (OPG) transcripts were exclusively upregulated in 67NR-derived B cells, particularly within TILs, peaking at day 11 and remaining elevated at day 21 (Fig. 7A). CD3^+^CD4^+^ T cells from 4T1 tumors expressed minimal levels of *Tnfrsf11b*, further emphasizing the differential immune programming induced by metastatic versus nonmetastatic tumor microenvironments (Fig. 7A). Consistent with the gene expression data, ELISA quantification of RANKL and OPG proteins in supernatants from *in vitro* restimulated lymphocytes confirmed distinct cytokine secretion profiles (Fig. 7B). Together, these results demonstrate that the tumor microenvironment itself harbors phenotypically distinct immune cell populations, with 4T1 tumors promoting a RANKL^+ ^CD3^+ ^CD4^+^ T cell phenotype and 67NR tumors sustaining OPG^+ ^CD19^+^ B cells.

### RANKL and OPG expression define divergent immune risk signatures in the BRCA-Basal subtype

**Figure 8.**
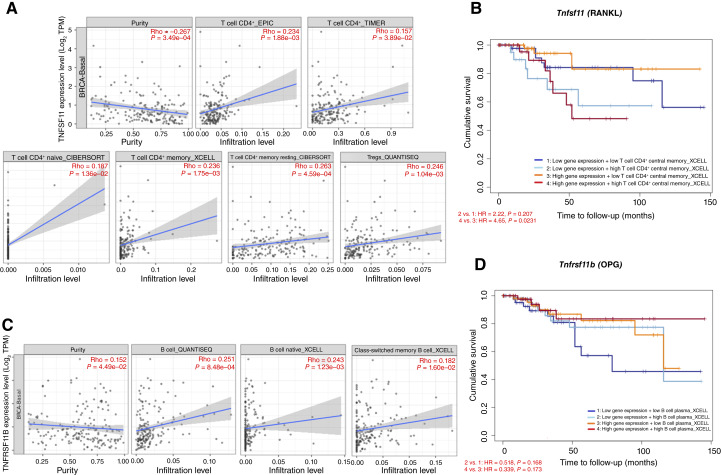
Divergent immune signatures defined by RANKL and OPG expression are associated with differential metastatic risk in BRCA-Basal breast tumors. **A,** Spearman correlation analysis between *TNFSF11* (RANKL) gene expression and CD4^+^ T cell subpopulations in BRCA-Basal tumors (*n* = 191, TCGA dataset). Significant positive correlations were observed with total CD4^+^ T cells (EPIC, TIMER), naive and memory subsets (CIBERSORT, XCELL), and Tregs (QUANTISEQ). An inverse correlation with tumor purity suggests an immune (rather than tumor) source of RANKL expression. **B,***Z*-score risk analysis identified high infiltration of CD4^+^ central memory T cells as significantly associated with increased clinical risk in the BRCA-Basal subtype (*Z* = 2.253; *P* < 0.05). **C,** Spearman correlations between *TNFRSF11B* (OPG) expression and B cell subpopulations in BRCA-Basal tumors. Significant positive associations were detected with total B cells (QUANTISEQ), naive B cells, and class-switched memory B cells (XCELL), alongside a negative correlation with tumor purity, reinforcing an immune origin for OPG expression. **D,***Z*-score risk analysis revealed that high plasma B cell infiltration was significantly associated with reduced clinical risk (*Z* = −2.078; *P* < 0.05), implicating a protective immune profile related to OPG expression. Data were obtained from the TCGA cohort using TIMER 2.0 and analyzed through multiple deconvolution algorithms (EPIC, TIMER, CIBERSORT, XCELL, QUANTISEQ). **A** and **D,** Correlation analyses between RANKL (*Tnfsf11*) or OPG (*Tnfrsf11b*) expression and immune cell infiltration across the BRCA-Basal TCGA cohort (*n* = 191) were performed using the TIMER, EPIC, CIBERSORT, XCELL, and QUANTISEQ deconvolution platforms. Statistical significance was determined by Spearman’s rank correlation test. **B** and **C,** Clinical risk associations for each immune subset were computed using *Z*-score analysis (two-tailed). Significance thresholds: *P* < 0.05.

To assess the clinical relevance of the immunophenotypes identified in our preclinical model, we conducted a comprehensive analysis of RANKL (*Tnfsf11*) and OPG (*Tnfrsf11b*) expression in relation to CD4^+^ T and B cell subpopulations across breast cancer subtypes using the TIMER database, with a focused evaluation of the BRCA-Basal group (*n* = 191), which includes TNBC. Spearman correlation analyses revealed statistically significant positive correlations between RANKL expression within the tumor microenvironment and total infiltrating CD4^+^ T cells (EPIC, ρ = 0.234, *P* = 1.88e−03; TIMER, ρ = 0.157, *P* = 3.89e−02), naive CD4^+^ T cells (CIBERSORT, ρ = 0.187, *P* = 1.36e−02), and memory CD4^+^ T cells (XCELL, ρ = 0.236, *P* = 1.75e−03). Additionally, we observed robust correlations between resting CD4^+^ memory T cells (CIBERSORT, ρ = 0.263, *P* = 4.59e−04) and regulatory T cells (Treg; QUANTISEQ, ρ = 0.246, *P* = 1.04e−03; Fig. 8A). These associations suggest that higher RANKL expression correlates with increased infiltration of multiple CD4^+^ T cell phenotypes. Importantly, the inverse correlation between RANKL expression and tumor purity (ρ = −0.267, *P* = 3.49e−04) supports the hypothesis that RANKL expression is primarily of immune rather than tumor origin, reinforcing its role in shaping the immunologic microenvironment of BRCA-Basal tumors (Fig. 8A). Notably, *Z*-score risk analysis identified CD4^+^ central memory T cells as the only subset in which increased presence, associated with high RANKL expression, was significantly associated with an elevated clinical risk (*Z* = 2.253; *P* < 0.05; Fig. 8B). This suggests a specific role for RANKL-expressing CD4^+^ T cells in the metastatic progression of TNBC. In contrast to RANKL, the expression of the OPG gene (*Tnfrsf11b*) is associated with a protective immunologic signature in BRCA-Basal tumors. Notably, *Z*-score analysis revealed that high infiltration of plasma B cells (*Z* = −2.078; *P* < 0.05), accompanied by high OPG expression, was significantly associated with reduced metastatic risk (Fig. 8C). This also aligns with the experimental results. In the BRCA-Basal cohort, OPG expression was positively correlated with B-cell infiltration analyzed on different platforms, such as QUANTISEQ for total B cells (ρ = 0.251, *P* = 8.48e−04), XCELL for naive B cells (ρ = 0.243, *P* = 1.23e−03), and class-switched memory B cells (ρ = 0.182, *P* = 1.60e−02; Fig. 8D). Additionally, concordant with the RANKL data shown above, there was a negative correlation between OPG expression and tumor purity (ρ = –0.152, *P* = 4.49e−02), reinforcing the notion that OPG expression originates primarily from immune infiltrates (Fig. 8D). Taken together, these findings highlight opposing immune axes in BRCA-Basal tumors: a RANKL^+ ^CD4^+^ T-cell phenotype associated with increased clinical risk and an OPG^+ ^B cell phenotype, particularly enriched in plasma B cells, associated with reduced metastatic risk. The overlap between these bioinformatic findings and our preclinical *in vivo* data underscores the translational potential of these immune phenotypes as prognostic biomarkers for bone metastasis in TNBC.

### Human TNBC samples exhibit contrasting infiltration patterns of RANKL^+^ and OPG^+^ lymphocytes predictive of bone metastatic risk

**Figure 9.**
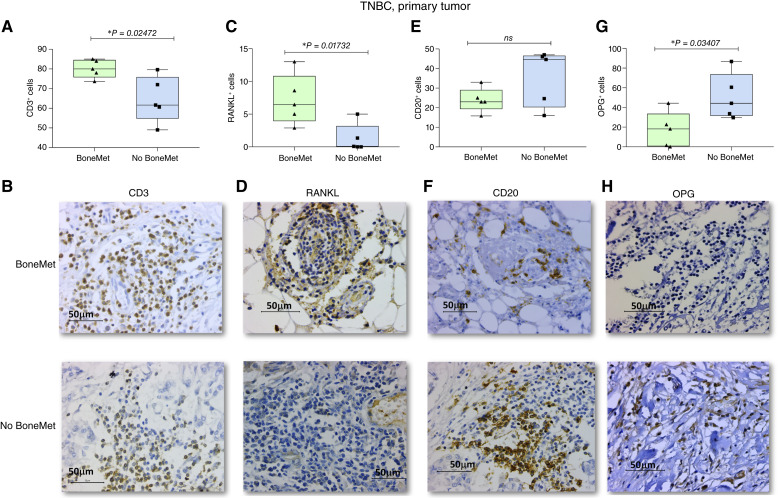
Opposing infiltration patterns of RANKL^+^ and OPG^+^ lymphocytes in primary tumors of patients with TNBC with or without bone metastases. **A,** Quantification of tumor-infiltrating CD3^+^ T cells in primary tumor samples from patients with TNBC who later developed BoneMet (*n* = 5) vs. those who remained No BoneMet (*n* = 5). Data are shown as box and whisker plots with individual values, and statistical comparison was performed by unpaired two-tailed Student *t* test (*P* = 0.0247); variance homogeneity was verified by F test (*P* = 0.0984). **B,** Representative IHC images of CD3^+ ^T cells in primary tumors from each patient group. **C,** Quantification of RANKL^+^ lymphocytes in the same cohort, also expressed as box and whisker plots with overlaid points. Statistical analysis: Mann–Whitney U test (*P* = 0.0278) and confirmatory unpaired two-tailed *t* test (*P* = 0.0173), with F test confirming homogeneity of variance (*P* = 0.2850). **D,** Representative images of RANKL staining in tumor sections from BoneMet and No BoneMet patients. **E,** Quantification of CD20^+^ B cells in the same tumor samples. Although not statistically significant (*P* = 0.1323) by unpaired two-tailed *t* test, a higher median infiltration was observed in the No BoneMet group, and F test for variance (*P* = 0.1287). **F,** Representative IHC images of CD20 staining showing differences in localization and density between groups. **G,** Quantification of OPG^+^ lymphocytes in No BoneMet tumors compared with BoneMet. Median values were 50.95% (No BoneMet) and 17.31% (BoneMet). Statistical analysis: unpaired two-tailed *t* test (*P* = 0.0341) with variance verified by F test (*P* = 0.6365); the Mann–Whitney U test showed a consistent trend (*P* = 0.0556). **H,** Representative images of OPG^+^ cells from each group. Statistical assumptions were verified using the F test for variance homogeneity. Due to the limited sample size, nonparametric tests were prioritized, and parametric results were used as confirmatory evidence.

To evaluate whether the immune phenotypes identified in murine models are also clinically relevant in human breast cancer, we analyzed primary tumor samples from patients diagnosed with TNBC, stratified into two groups: those who either presented with stage IV disease involving the bone or developed bone metastases during clinical follow-up (BoneMet) and those who remained free of metastatic disease for at least 5 years (No BoneMet). First, we evaluated the overall infiltration of CD3^+^ T lymphocytes in these samples. Quantification of CD3^+^ T cells revealed significantly higher infiltration in primary tumors from BoneMet patients than in the No BoneMet controls (median: 80.13% vs. 64.60%; Fig. 9A and B). Variance analysis using the F test showed no significant difference between the groups (*P* = 0.0984), supporting the reliability of the *t* test results. In both the BoneMet and No BoneMet groups, CD3^+^ T lymphocytes were readily detected within the tumor stroma, exhibiting a similar spatial distribution and staining intensity (Fig. 9B). We also assessed the presence of RANKL^+^ lymphocytes in this cohort. IHC analysis revealed significantly higher infiltration of RANKL^+^ cells in primary tumors from BoneMet patients than in the No BoneMet controls (Fig. 9C and D). Quantification demonstrated mean frequencies of 7.192% in the BoneMet group and 1.286% in the No BoneMet group (Fig. 9C). Owing to the small sample size (*n* = 5 per group), statistical analysis was initially performed using the nonparametric Mann–Whitney U test, which identified a significant difference (*P* = 0.0278). To validate the consistency of this finding, an unpaired two-tailed *t* test was conducted, confirming statistical significance (*P* = 0.0173; mean difference = −5.906 ± 1.975; 95% CI = −10.46 to −1.352; *R*^2^ = 0.5278). The F test for variance homogeneity indicated no significant difference in variances (*P* = 0.2850), validating the use of the *t* test (Fig. 9C). Histologically, RANKL staining was localized predominantly to lymphocyte-like cells distributed within the tumor microenvironment, exhibiting strong cytoplasmic and membranous signals (Fig. 9D). In contrast, tumors from patients with No-BoneMet displayed sparse RANKL expression, with markedly fewer stained cells and reduced signal intensity (Fig. 9D). The proportion of CD20^+^ B cells within the two groups (Fig. 9E) did not reach statistical significance (*P* = 0.1323, unpaired *t* test). However, a clear trend was observed for increased infiltration of CD20^+^ B cells in tumors from patients who did not develop bone metastases (median: 35.66% vs. 23.97%; Fig. 9E). Homogeneity of variance for CD20^+^ cells was confirmed (F test, *P* = 0.1287), and both parametric and nonparametric analyses supported the same directional effect (Fig. 9E). Representative IHC staining images further illustrated the differential localization and abundance of CD20^+^ cells between the two patient groups (Fig. 9F). Conversely, quantification of OPG^+^ cells revealed significantly higher infiltration in tumors from patients who remained free of bone metastases (No BoneMet; median: 50.95%) than in those who progressed to skeletal dissemination (BoneMet; 17.31%), as determined by an unpaired two-tailed *t* test (*P* = 0.0341; Fig. 9G). Homogeneity of variance was confirmed (F test, *P* = 0.6365), and the observed difference was biologically meaningful despite the limited sample size (*n* = 5 per group). The Mann–Whitney U test also indicated a consistent trend (*P* = 0.0556), with higher median values of OPG^+^ cells in the No BoneMet group (44.18%) than in the BoneMet group (18.18%). Representative IHC images illustrate this difference (Fig. 9H). These findings are consistent with our preclinical data in murine models, in which OPG-expressing CD19^+^ B cells suppressed osteoclastogenesis and limited bone metastatic colonization, whereas RANKL-expressing CD4^+^ T cells facilitated premetastatic niche formation. Collectively, these results strengthen the translational relevance of CD20^+ ^OPG^+^ B cells as potential immune effectors capable of antagonizing bone metastasis in breast cancer. The coexistence of RANKL^+^ T cells and OPG^+^ B cells within the tumor microenvironment suggests a functional balance between risk-promoting and protective lymphocyte subsets, offering a novel prognostic framework for stratifying patients based on their propensity to develop skeletal metastases.

## Discussion

This study revealed a previously unrecognized immunomodulatory role of CD19^+^ B cells in nonmetastatic breast cancer, demonstrating their capacity to regulate bone remodeling and limit tumor progression. Traditionally viewed as secondary or passive components within the tumor-immune microenvironment, our findings show that CD19^+^ B cells acquire a regulatory phenotype when primed by nonmetastatic 67NR tumors. This phenotype is characterized by increased secretion of OPG, suppression of protumoral and premetastatic activity of 4T1^+^ RANKL-expressing T cells, and a consequent reduction in tumor burden and bone metastases. The absence of this phenotype in B cells from T cell–deficient tumor-bearing hosts suggests that this effector program is T cell–dependent, likely requiring early B cell–T cell interactions during tumor priming. These insights suggest that CD19^+^ B cells are active regulators of bone colonization, highlighting the functional cross-talk between adaptive immune subsets in controlling bone-associated tumor dissemination.

The concept of an immune-driven premetastatic bone niche was originally proposed by our group (25), which demonstrated that tumor-specific CD3^+ ^RANKL^+^ T cells were sufficient to induce osteolytic remodeling and establish a permissive bone environment before detectable tumor colonization. Building on this foundation, the present study identifies a counter-regulatory mechanism mediated by OPG-producing CD19^+^ B cells that functionally restrain RANKL^+^ T cells and prevent osteolytic progression. Although a tumor-intrinsic pathway of niche formation through G-CSF–dependent vascular remodeling has been described (32), our data highlight an adaptive immune mechanism acting locally within the BM and tumor microenvironment. Together, these immune- and tumor-derived pathways represent complementary yet distinct routes of premetastatic niche formation that converge to determine the osteoimmune landscape and metastatic susceptibility.

It is well established that tumor-infiltrating B cells play dual roles in tumor biology, either by promoting or inhibiting tumor growth, depending on local factors such as antigen exposure, cytokine milieu, and spatial localization (33–38). For example, regulatory B cells (Breg) facilitate immune evasion via IL-10, TGFβ, and PD-L1, thereby suppressing cytotoxic T-cell responses (36, 39–41). In contrast, antigen-experienced B cells, including memory B cells and plasma cells, contribute to antitumor immunity through antibody production, tertiary lymphoid structure formation, and antigen presentation to T cells (42–48). Single-cell transcriptomic studies have further identified tumor-associated atypical B cells marked by *FCRL4* expression, enriched for MHC-II and CD4^+^ T-cell interactions, and precursors of antibody-secreting cells (49–51). Although our study did not profile the transcriptional signatures of OPG-producing B cells, the phenotype observed (CD19^+ ^IgD^+ ^IgM^+ ^CD138^−^) in the 67NR model does not correspond to classical Bregs or atypical B cells but suggests a distinct regulatory subset specialized in bone remodeling.

B cell–derived OPG is recognized as vital in physiologic bone homeostasis, and its downregulation is linked to bone-destructive diseases such as chronic periodontitis and HIV infection (8, 14, 15). Our data suggest that the tumor influences B cell phenotypes, potentially disrupting bone regulatory mechanisms to facilitate metastasis. Recent findings have described CD19^+ ^LAG-3^+^ B cells in murine 4T1 and EO771 models and human TNBC, which are enriched in tumor-draining LNs and display signatures of proliferation (52). Notably, high *LAG-3*^*+*^ B-cell transcript expression correlates with longer progression-free survival in TNBC, indicating a potential prognostic role (52). These observations align with clinical studies associating high densities of CD20^+^ B cells in sentinel LNs (including tumor-free nodes) with improved disease-free survival across TNBC, basal-like, and HER2-enriched breast cancer subtypes (53–58). Conversely, the expansion of circulating immunosuppressive CD19^+ ^CD24^+ ^CD38^+^ Bregs is linked to poor prognosis, underscoring the functional polarity of B cell subsets in breast cancer (59). Even at early disease stages, B cells may exert protective surveillance, as the absence of CD20^+^ B cells in benign lobular units correlates with an increased malignancy risk (33, 55).

OPG, like RANKL, is a multifunctional molecule that is critical beyond its recognized role as a passive decoy receptor. It dynamically modulates the homeostasis of breast tissue, thymus, intestines, bone, and the immune system (6, 60–62). In the BM, mature B cells are a significant source of OPG, relying on CD40-CD40L T cell interactions for this function (8). Mice deficient in CD40, CD40L, or B cells exhibit reduced OPG levels and BMD, which can be rescued by B cell reconstitution (8). Our findings parallel these physiologic mechanisms: In 67NR-bearing mice, CD19^+ ^IgD^+ ^IgM^+ ^CD138^−^ B cells require T cell licensing to acquire and sustain an OPG-producing phenotype. Silencing OPG in these B cells abrogates their regulatory capacity, reinstates osteoclastogenesis, and increases 4T1-driven bone metastasis. Moreover, physiologic RANKL-OPG signaling operates in a highly localized manner, as demonstrated by *in vivo* studies showing that tissue-specific, rather than circulating, OPG is indispensable for maintaining bone and immune homeostasis RANKL-OPG signaling is locally orchestrated, as demonstrated by *in vivo* studies showing that tissue-specific, but not serum-specific, OPG is essential for maintaining bone and immune homeostasis (6). Conditional OPG deletion in OBs, thymic epithelial cells, or intestinal microfold cells causes osteopenia and immune dysregulation despite normal serum OPG levels, underscoring the critical importance of local OPG production.

It is essential to emphasize that OPG does not transcriptionally repress RANKL but instead neutralizes its biological activity through competitive binding (63). The reduced levels of soluble RANKL detected in our assays represent RANKL secreted by T cells derived from the spleen and BM after *in vitro* restimulation with 4T1 tumor–derived sAgs. Thus, these findings reveal a functional modulation of tumor-specific T-cell responses in the presence of OPG-producing B cells, rather than a direct molecular suppression of RANKL gene expression. Consistent with this, our previous work demonstrated that 67NR-bearing mice display increased OB numbers and enhanced bone formation (30), indicating that nonmetastatic tumors generate an osteoanabolic bone microenvironment. It is therefore plausible that OPG^+^ B cells and OB-lineage cells cooperate to reestablish the RANKL-OPG balance and restrain osteoclastogenic activity. Local enrichment of OPG may influence paracrine signaling among T cells, B cells, and stromal/OB cells, collectively contributing to the attenuation of tumor-induced bone resorption. Future studies will be required to delineate these cellular interactions and to determine whether B cell–derived OPG functions as a regulatory bridge between adaptive immune responses and bone remodeling.

The present study was designed to establish functional causality rather than to exhaustively phenotype every immune subset in the tumor microenvironment. Nonetheless, multiple lines of evidence indicate that these cells act as active regulatory effectors rather than passive bystanders. First, in SCID recipients, in which all lymphocytes are experimentally introduced, B cell–derived OPG was strictly required to suppress antigen-driven RANKL measurements, preserve trabecular bone architecture, and prevent BM metastasis. Second, B cells isolated from T cell–deficient donors failed to acquire the OPG^+^ phenotype, indicating that T cell help is necessary for the *in vivo* licensing of this subset of B cells. Although the precise molecular pathway that induces OPG in tumor-primed B cells remains to be defined, these data position OPG^+^ B cells as active mediators of osteoimmune protection and identify T cell–B cell cross-talk as a critical upstream licensing step.

In our model, local OPG production also limits primary 4T1 tumor growth, consistent with the expression of RANK by 4T1 cells of mammary epithelial origin. These findings expand the current understanding of OPG biology, highlighting that its function is highly context-dependent. Epidemiologic studies have shown that elevated systemic OPG correlates with increased risk in estrogen receptor–negative breast cancer, possibly due to its ability to inhibit TRAIL-mediated apoptosis, whereas high local OPG expression is associated with protection in estrogen receptor–positive tumors, particularly in premenopausal women (64, 65). Therefore, the biological outcome of OPG depends on its spatial and temporal distribution, with systemic OPG potentially favoring tumor persistence, whereas locally produced OPG supports tissue-specific homeostasis and restrains osteolytic and metastatic processes.

The use of B cells as an immunotherapeutic tool necessitates defining the optimal therapeutic window. Kinetic analyses indicated that the regulatory phenotype of 67NR-primed CD19^+ ^OPG^+^ B cells is most effective when adoptively transferred early during 4T1 tumor progression. Mice receiving B cells at tumor inoculation exhibited marked suppression of primary tumor growth and decreased RANKL expression in the BM and within the tumor microenvironment, but no detectable modulation in draining LNs at the analyzed time points. Transfers performed on day 7 after inoculation, when the premetastatic niche was already established, conferred only partial protection, whereas those on day 17, after metastatic dissemination, failed to limit disease progression. These findings emphasize the spatial and temporal constraints of the protective effects mediated by CD19^+ ^OPG^+^ B cells, which require early engagement with both bone and tumor compartments to exert their full regulatory potential. Consistent with this temporal dependency, the spatial restriction of these effects reinforces the concept that OPG^+^ B cells act primarily within the osteoimmune microenvironment. Clonogenic assays confirmed that although early transfer of 67NR-derived B cells completely abrogated BM metastases, metastatic colonization in visceral organs such as the lung and liver remained unaffected (Supplementary Fig. S2). This finding indicates that the protective effect of OPG-producing B cells is locally confined to the bone niche, in which these cells can directly counteract preosteolytic remodeling. The absence of an important impact on pulmonary metastases further underscores the tissue specificity of OPG-mediated regulation, consistent with the concept that local, rather than systemic, OPG production governs the balance between osteolytic and osteoanabolic immune responses.

From a translational standpoint, these results highlight a previously unrecognized layer of adaptive immune control over the RANKL–RANK–OPG axis. Unlike the uniform systemic neutralization achieved by Denosumab (66, 67), OPG production by B cells occurs in anatomically restricted sites, in which it may enable context-dependent modulation of both bone resorption and local immune responses. This spatially confined expression likely contributes to the preservation of skeletal integrity and the attenuation of pro-osteolytic immune phenotypes in the tumor milieu. Although the precise cellular interactions mediating these effects remain to be defined, the observed correlation between B cell–derived OPG expression, reduced RANKL levels, and diminished metastatic skeletal burden supports the concept that immune-derived OPG acts as part of a coordinated feedback network linking adaptive immunity, tumor regulation, and bone remodeling. Future studies will be required to elucidate the molecular and temporal cues that define this protective B cell phenotype in both osseous and tumor contexts.

Transcriptomic analysis of the BRCA-Basal subtype, which includes most TNBC cases in The Cancer Genome Atlas (TCGA) dataset, revealed that RANKL expression correlates with increased CD4^+^ T-cell infiltration and lower tumor purity, indicating immune cell origin and higher metastatic risk. Conversely, OPG expression is associated with plasma and memory B cells and reduces metastatic risk. IHC analysis of human TNBC tumors supports the hypothesis that patients without bone metastases show a higher infiltration of OPG^+^ lymphocytes, whereas those progressing to bone metastases exhibit increased RANKL^+^ lymphocytes. Although limited by sample size and the inability to precisely identify lymphocyte subsets expressing RANKL, these observations align with murine data, confirming that RANKL^+^ lymphocytes are predominantly CD3^+ ^CD4^+^ T cells.

The correlation of RANKL and OPG expression with memory T and B cell subsets in the BRCA-Basal cohort suggests that adaptive immune memory may play a pivotal role in sustaining osteoimmune balance. These associations imply that long-lived memory lymphocytes could preserve the regulatory equilibrium between RANKL^+ ^CD4^+^ T cells and OPG^+^ B cells, contributing to long-term protection against osteolytic progression. Future studies will aim to characterize these subsets phenotypically and functionally, determining whether memory-derived OPG^+^ B cells and RANKL^+^ T cells represent durable reservoirs of immune regulation in breast cancer. Together, the murine and human datasets converge on the same adaptive immune mechanism, wherein the balance between RANKL^+ ^CD4^+^ T cells and OPG^+ ^B cells dictates bone metastatic outcomes. Although our functional murine experiments defined causality, the clinical analyses validate the presence of corresponding immune signatures in the primary tumors, stratifying patients who are most likely to progress with or without bone metastasis. Future work integrating phenotypic and transcriptomic profiling of murine lymphocyte subsets will further align these datasets and refine the mechanistic basis of this immune regulation.

Although this study focused on TNBC, other breast cancer subtypes may share similar mechanisms of metastasis (1). Extending the investigation of RANKL-OPG immune phenotypes to other subtypes is essential to determine whether this axis represents a generalizable metastatic mechanism in breast cancer. Given their early appearance in primary tumors, these insights could guide the development of stratified prognostic tools and personalized immunotherapies that target skeletal integrity and metastatic progression. Future prospective studies with larger, more diverse cohorts are needed to validate these immune biomarkers and explore their clinical utility in guiding bone metastasis interventions.

## Supplementary Material

Supplementary Figure 1 Kinetics of OPG secretion by bone marrow CD19^+^ B cells from 67NR tumor–bearing mice.

Supplementary Figure 2 CD19^+^ B cells from 67NR tumor-bearing mice suppress osteolytic and metastatic disease in immunocompetent BALB/c mice.

Supplementary Figure 3 The regulatory phenotype by CD19^+^ B cells from 67NR tumor-bearing mice does not suppress spleen, liver, and lung metastases.

Supplementary Figure 4 Purity assessment of CD19^+^ B cells used for adoptive transfer experiments.

Supplementary Figure 5 Sorting of tumor-infiltrating CD4^+^ T and CD19^+^ B cells from 4T1 and 67NR tumor-bearing mice

Supplementary Table 1 Research Resource Identifiers (RRIDs) for reagents, antibodies, cell lines, kits, equipment, and software used in this study

## Data Availability

The transcriptomic data from human primary breast tumors analyzed in this study are publicly available through the TCGA (TCGA-BRCA) repository (https://portal.gdc.cancer.gov/). Experimental datasets generated during this study, including flow cytometry, qPCR, micro-CT imaging, and histomorphometry, are available from the corresponding author upon reasonable request. All relevant quantitative data supporting the findings are also included in the main text and supplementary materials. Human IHC data obtained from FFPE tumor specimens are not publicly available due to ethical and privacy restrictions but may be accessed upon request, subject to approval by the institutional ethics committee and compliance with patient confidentiality agreements.
