# Supplementary material for: OPG-Producing B Cells and RANKL-Expressing T Cells Define Immune Signatures Predictive of Bone Metastases in Breast Cancer
Source: Cancer Res Commun. 2026 Jan 13;6(1):85–104. doi: 10.1158/2767-9764.CRC-25-0696 (PMC12795788; doi:10.1158/2767-9764.CRC-25-0696)
Supplement: Supplementary Figure 1 — Kinetics of OPG secretion by bone marrow CD19+ B cells from 67NR tumor–bearing mice. [file crc-25-0696_supplementary_figure_1_suppsf1.pptx]

## Slide 1
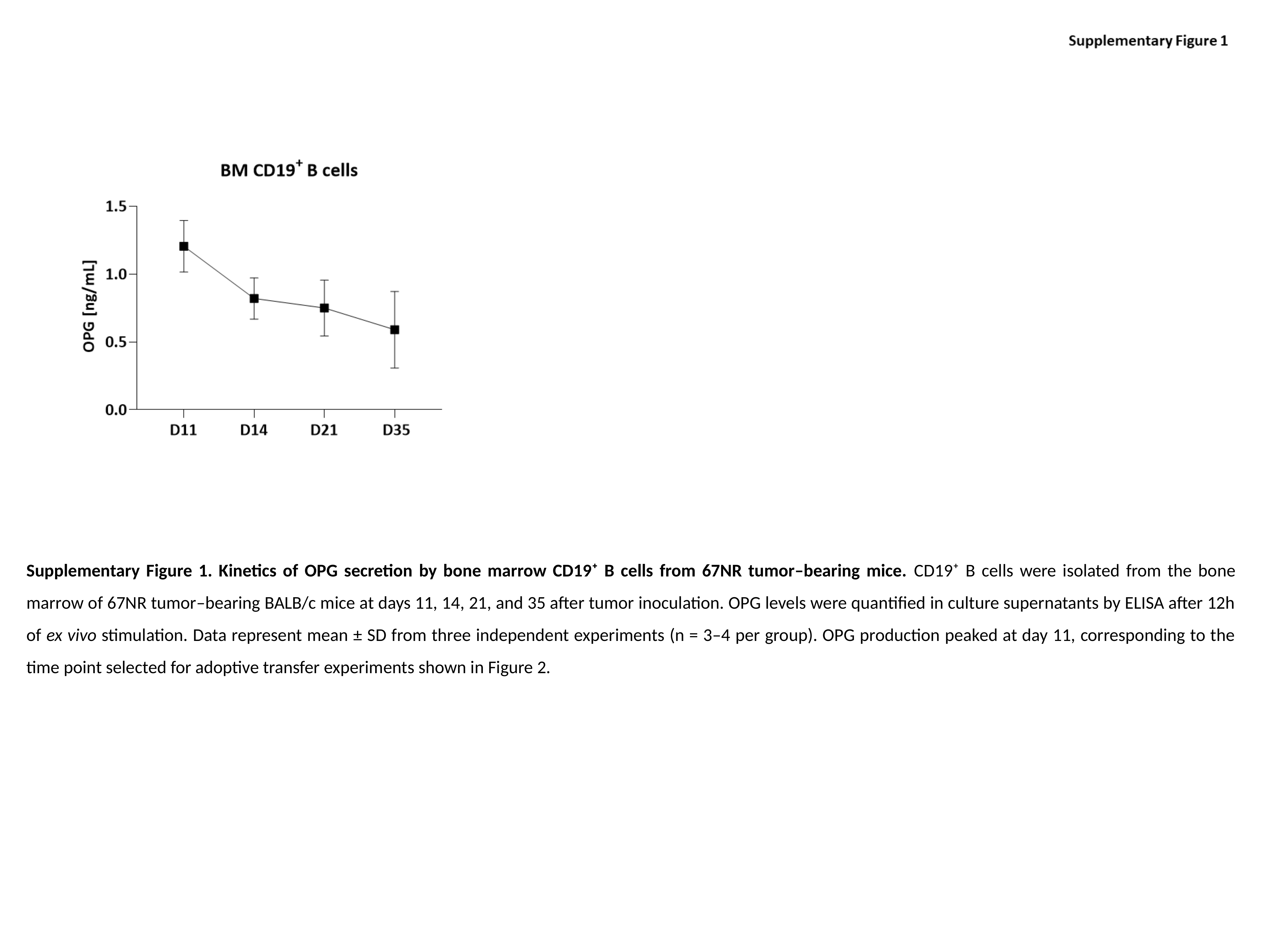

Supplementary Figure 1. Kinetics of OPG secretion by bone marrow CD19⁺ B cells from 67NR tumor–bearing mice. CD19⁺ B cells were isolated from the bone marrow of 67NR tumor–bearing BALB/c mice at days 11, 14, 21, and 35 after tumor inoculation. OPG levels were quantified in culture supernatants by ELISA after 12h of ex vivo stimulation. Data represent mean ± SD from three independent experiments (n = 3–4 per group). OPG production peaked at day 11, corresponding to the time point selected for adoptive transfer experiments shown in Figure 2.
