# Supplementary material for: OPG-Producing B Cells and RANKL-Expressing T Cells Define Immune Signatures Predictive of Bone Metastases in Breast Cancer
Source: Cancer Res Commun. 2026 Jan 13;6(1):85–104. doi: 10.1158/2767-9764.CRC-25-0696 (PMC12795788; doi:10.1158/2767-9764.CRC-25-0696)
Supplement: Supplementary Figure 2 — CD19+ B cells from 67NR tumor-bearing mice suppress osteolytic and metastatic disease in immunocompetent BALB/c mice. [file crc-25-0696_supplementary_figure_2_suppsf2.pptx]

## Slide 1
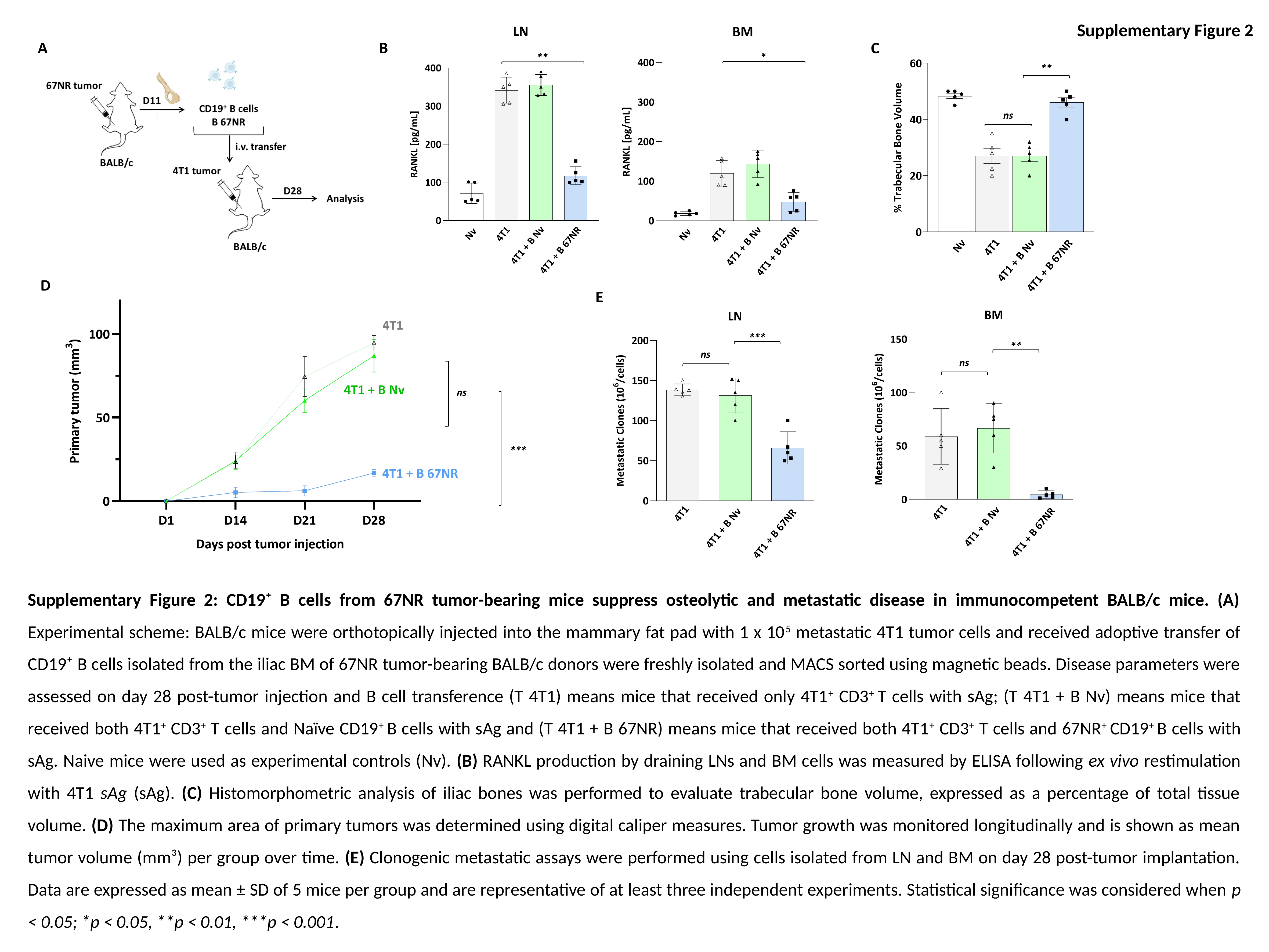

Supplementary Figure 2
Supplementary Figure 2: CD19⁺ B cells from 67NR tumor-bearing mice suppress osteolytic and metastatic disease in immunocompetent BALB/c mice. (A) Experimental scheme: BALB/c mice were orthotopically injected into the mammary fat pad with 1 x 10^5^ metastatic 4T1 tumor cells and received adoptive transfer of CD19⁺ B cells isolated from the iliac BM of 67NR tumor-bearing BALB/c donors were freshly isolated and MACS sorted using magnetic beads. Disease parameters were assessed on day 28 post-tumor injection and B cell transference (T 4T1) means mice that received only 4T1+ CD3+ T cells with sAg; (T 4T1 + B Nv) means mice that received both 4T1+ CD3+ T cells and Naïve CD19+ B cells with sAg and (T 4T1 + B 67NR) means mice that received both 4T1+ CD3+ T cells and 67NR+ CD19+ B cells with sAg. Naive mice were used as experimental controls (Nv). (B) RANKL production by draining LNs and BM cells was measured by ELISA following ex vivo restimulation with 4T1 sAg (sAg). (C) Histomorphometric analysis of iliac bones was performed to evaluate trabecular bone volume, expressed as a percentage of total tissue volume. (D) The maximum area of primary tumors was determined using digital caliper measures. Tumor growth was monitored longitudinally and is shown as mean tumor volume (mm³) per group over time. (E) Clonogenic metastatic assays were performed using cells isolated from LN and BM on day 28 post-tumor implantation. Data are expressed as mean ± SD of 5 mice per group and are representative of at least three independent experiments. Statistical significance was considered when p < 0.05; *p < 0.05, **p < 0.01, ***p < 0.001.
