# Supplementary material for: OPG-Producing B Cells and RANKL-Expressing T Cells Define Immune Signatures Predictive of Bone Metastases in Breast Cancer
Source: Cancer Res Commun. 2026 Jan 13;6(1):85–104. doi: 10.1158/2767-9764.CRC-25-0696 (PMC12795788; doi:10.1158/2767-9764.CRC-25-0696)
Supplement: Supplementary Figure 3 — The regulatory phenotype by CD19+ B cells from 67NR tumor-bearing mice does not suppress spleen, liver, and lung metastases. [file crc-25-0696_supplementary_figure_3_suppsf3.pptx]

## Slide 1
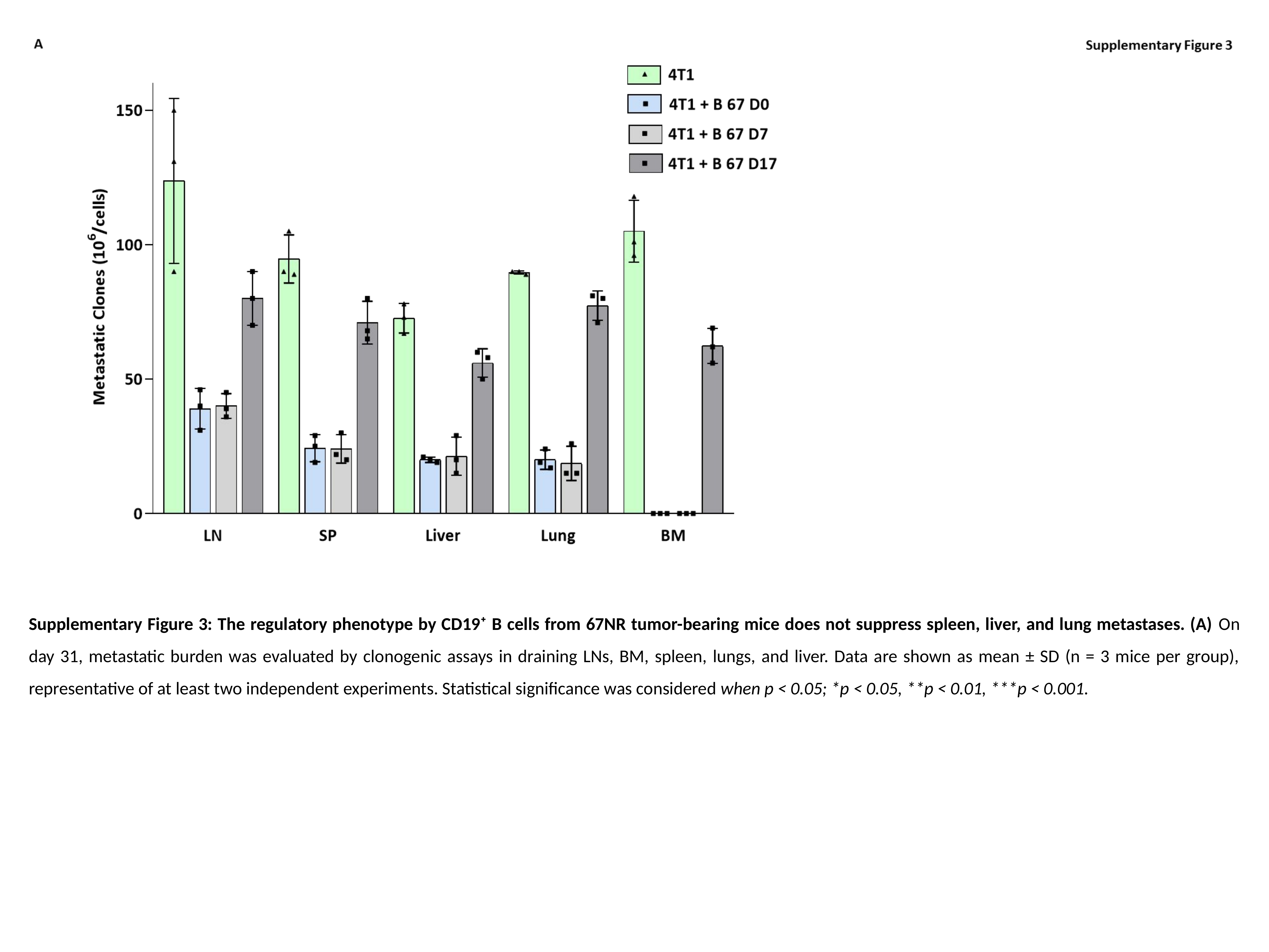

Supplementary Figure 3: The regulatory phenotype by CD19⁺ B cells from 67NR tumor-bearing mice does not suppress spleen, liver, and lung metastases. (A) On day 31, metastatic burden was evaluated by clonogenic assays in draining LNs, BM, spleen, lungs, and liver. Data are shown as mean ± SD (n = 3 mice per group), representative of at least two independent experiments. Statistical significance was considered when p < 0.05; *p < 0.05, **p < 0.01, ***p < 0.001.
