# Supplementary material for: OPG-Producing B Cells and RANKL-Expressing T Cells Define Immune Signatures Predictive of Bone Metastases in Breast Cancer
Source: Cancer Res Commun. 2026 Jan 13;6(1):85–104. doi: 10.1158/2767-9764.CRC-25-0696 (PMC12795788; doi:10.1158/2767-9764.CRC-25-0696)
Supplement: Supplementary Figure 4 — Purity assessment of CD19+ B cells used for adoptive transfer experiments. [file crc-25-0696_supplementary_figure_4_suppsf4.pptx]

## Slide 1
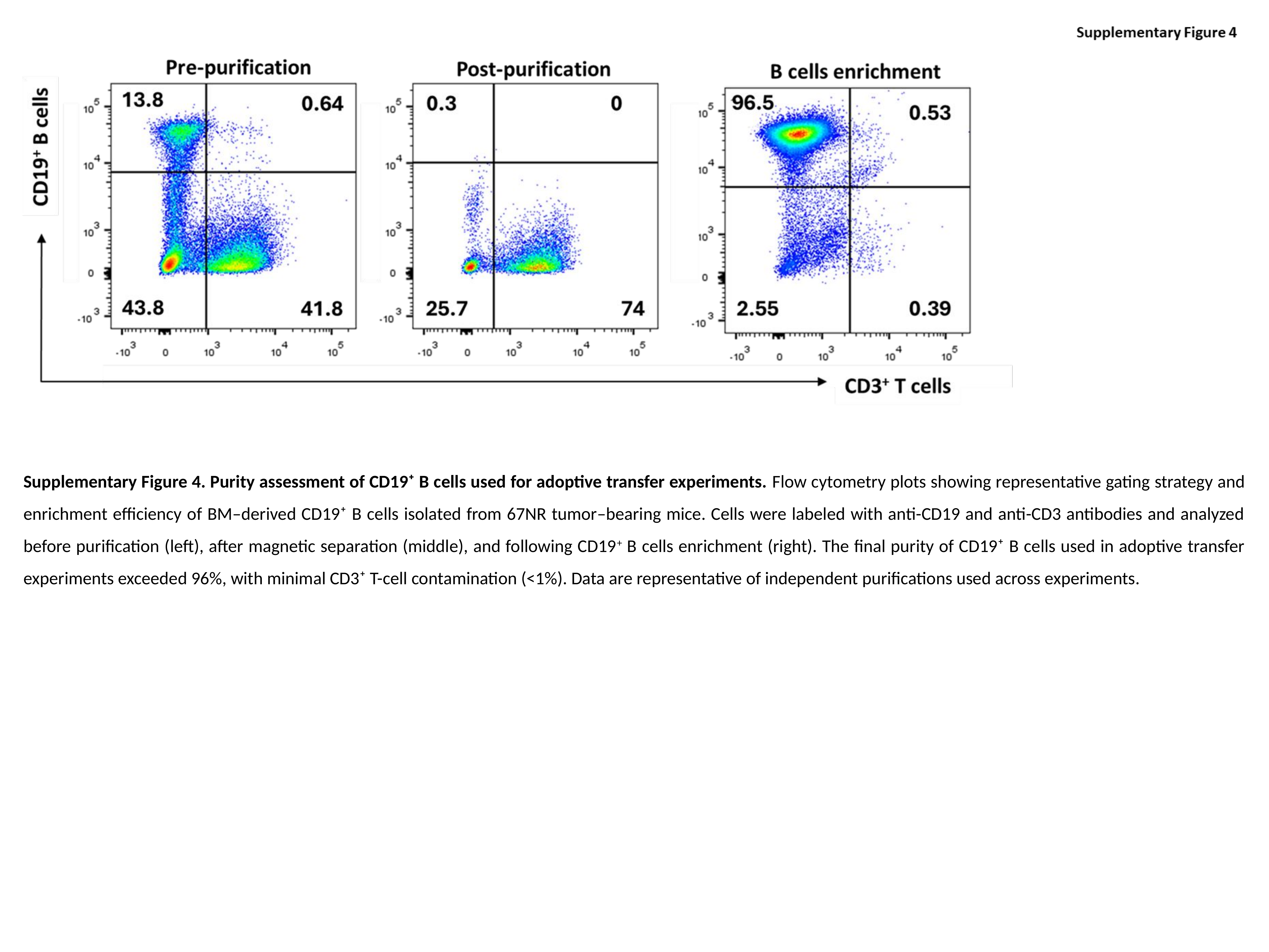

Supplementary Figure 4. Purity assessment of CD19⁺ B cells used for adoptive transfer experiments. Flow cytometry plots showing representative gating strategy and enrichment efficiency of BM–derived CD19⁺ B cells isolated from 67NR tumor–bearing mice. Cells were labeled with anti-CD19 and anti-CD3 antibodies and analyzed before purification (left), after magnetic separation (middle), and following CD19+ B cells enrichment (right). The final purity of CD19⁺ B cells used in adoptive transfer experiments exceeded 96%, with minimal CD3⁺ T-cell contamination (<1%). Data are representative of independent purifications used across experiments.
