# Supplementary material for: OPG-Producing B Cells and RANKL-Expressing T Cells Define Immune Signatures Predictive of Bone Metastases in Breast Cancer
Source: Cancer Res Commun. 2026 Jan 13;6(1):85–104. doi: 10.1158/2767-9764.CRC-25-0696 (PMC12795788; doi:10.1158/2767-9764.CRC-25-0696)
Supplement: Supplementary Figure 5 — Sorting of tumor-infiltrating CD4+ T and CD19+ B cells from 4T1 and 67NR tumor-bearing mice [file crc-25-0696_supplementary_figure_5_suppsf5.pptx]

## Slide 1
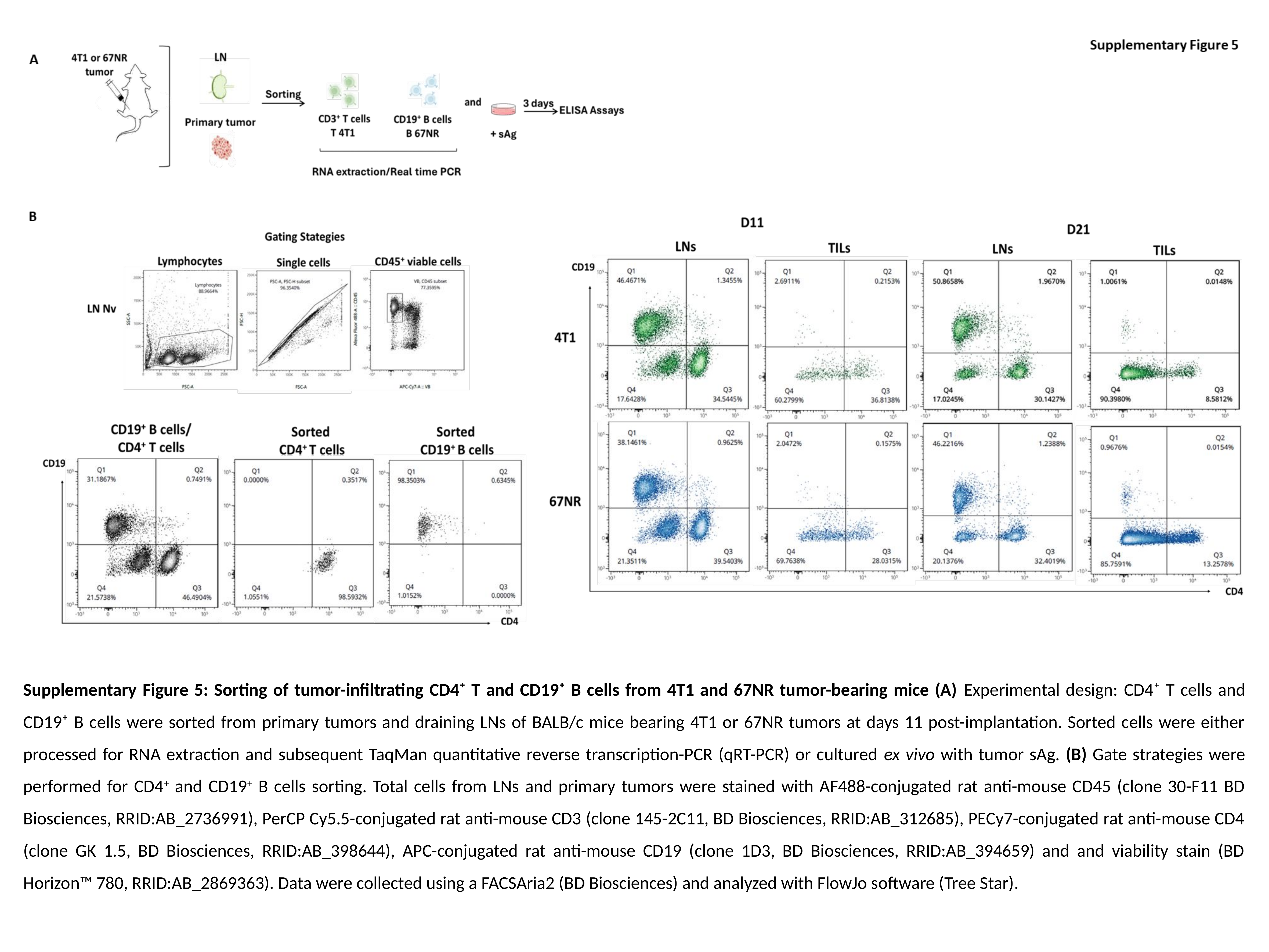

Supplementary Figure 5: Sorting of tumor-infiltrating CD4⁺ T and CD19⁺ B cells from 4T1 and 67NR tumor-bearing mice (A) Experimental design: CD4⁺ T cells and CD19⁺ B cells were sorted from primary tumors and draining LNs of BALB/c mice bearing 4T1 or 67NR tumors at days 11 post-implantation. Sorted cells were either processed for RNA extraction and subsequent TaqMan quantitative reverse transcription-PCR (qRT-PCR) or cultured ex vivo with tumor sAg. (B) Gate strategies were performed for CD4+ and CD19+ B cells sorting. Total cells from LNs and primary tumors were stained with AF488-conjugated rat anti-mouse CD45 (clone 30-F11 BD Biosciences, RRID:AB_2736991), PerCP Cy5.5-conjugated rat anti-mouse CD3 (clone 145-2C11, BD Biosciences, RRID:AB_312685), PECy7-conjugated rat anti-mouse CD4 (clone GK 1.5, BD Biosciences, RRID:AB_398644), APC-conjugated rat anti-mouse CD19 (clone 1D3, BD Biosciences, RRID:AB_394659) and and viability stain (BD Horizon™ 780, RRID:AB_2869363). Data were collected using a FACSAria2 (BD Biosciences) and analyzed with FlowJo software (Tree Star).
